# Probiotic-derived amphiphilic exopolysaccharide self-assembling adjuvant delivery platform for enhancing immune responses

**DOI:** 10.1186/s12951-024-02528-y

**Published:** 2024-05-19

**Authors:** Shouxin Sheng, Haochi Zhang, Xinyu Li, Jian Chen, Pu Wang, Yanchen Liang, Chunhe Li, Haotian Li, Na Pan, Xuemei Bao, Mengnan Liu, Lixia Zhao, Xiaoyan Li, Pingyuan Guan, Xiao Wang

**Affiliations:** 1 State Key Laboratory of Reproductive Regulation & Breeding of Grassland Livestock, School of Life Science, Inner Mongolia University, Hohhot, 010021 P.R. China; 2 JinYuBaoLing Biopharmaceutical Co. Ltd, Inner Mongolia, 010000 Hohhot P.R. China

**Keywords:** Immune-adjuvant, Exopolysaccharide, Self-assembly, Nanovaccine, Immune response

## Abstract

Enhancing immune response activation through the synergy of effective antigen delivery and immune enhancement using natural, biodegradable materials with immune-adjuvant capabilities is challenging. Here, we present NAPS^*L.p*^ that can activate the Toll-like receptor 4 (TLR4) pathway, an amphiphilic exopolysaccharide, as a potential self-assembly adjuvant delivery platform. Its molecular structure and unique properties exhibited remarkable self-assembly, forming a homogeneous nanovaccine with ovalbumin (OVA) as the model antigen. When used as an adjuvant, NAPS^*L.p*^ significantly increased OVA uptake by dendritic cells. In vivo imaging revealed prolonged pharmacokinetics of NAPS^*L. p*^-delivered OVA compared to OVA alone. Notably, NAPS^*L.p*^ induced elevated levels of specific serum IgG and isotype titers, enhancing rejection of B16-OVA melanoma xenografts in vaccinated mice. Additionally, NAPS^*L.p*^ formulation improved therapeutic effects, inhibiting tumor growth, and increasing animal survival rates. The nanovaccine elicited CD4^+^ and CD8^+^ T cell-based immune responses, demonstrating the potential for melanoma prevention. Furthermore, NAPS^*L.p*^-based vaccination showed stronger protective effects against influenza compared to Al (OH)_3_ adjuvant. Our findings suggest NAPS^*L.p*^ as a promising, natural self-adjuvanting delivery platform to enhance vaccine design across applications.

**Supplementary Information:**

The online version contains supplementary material available at 10.1186/s12951-024-02528-y.

## Introduction

Despite extensive efforts to enhance the effectiveness of vaccine adjuvants, the challenge of achieving sufficient immunogenicity persists [1, 2]. Conventional direct immunization with most antigens often leads to inadequate immune responses and rapid clearance of the antigens from the body [3]. To overcome these limitations, adjuvants are employed to enhance immunogenicity, reduce antigen dosage, prolong the prophylactic effect, and accelerate immune responses [4, 5]. However, only a limited number of adjuvants, such as Alum, MF59, virosomes, and montanide ISA-51, have received approval from the US Food and Drug Administration (FDA) [6–8]. Among these, particulate adjuvants based on aluminum salt precipitates (Alum) are the most commonly used, despite their relatively low potency and weak stimulation of cell-mediated immunity [9, 10]. Oil-in-water emulsions containing squalene (MF59 and AS03), as well as liposome-based adjuvant system AS01, are approved for human vaccines, they can occasionally lead to local and mild systemic adverse effects. These effects are generally short-lived and manageable, underscoring the importance of considering their benefits in improving vaccine efficacy and disease prevention [11, 12]. Due to those disadvantages, there is a pressing need to develop novel adjuvants that are both effective and non-toxic for use in organisms.

In recent decades, there have been significant efforts to improve vaccine efficacy through the use of delivery systems and enhanced adjuvants [13, 14]. Biodegradable polymers have emerged as a promising approach, allowing for efficient delivery of antigens encapsulated in nano-vehicles while enhancing immune responses and protecting antigens from degradation. One promising approach is the use of biodegradable polymers as adjuvants, which can encapsulate antigens in nano-vehicles for efficient delivery. These polymers not only enhance immune responses but also protect antigens from degradation. Thus, providing multiple benefits [15–18]. However, the heterogeneity and balance between immune enhancement and biological toxicity of chemical polymers remain challenges for their clinical application.

Natural productions, such as polysaccharides, proteins, and exosomes, as natural building blocks, offer a promising solution to overcome the limitations of synthetic polymers in immunologic adjuvant application [19–21]. Polysaccharides are most used in research due to the high productivity and biodegradability. Recently, polysaccharides-based adjuvants, such as Inulin, chitosan, and alginate, have been reported to possess favorable characteristics of low toxicity and biodegradability [22]. However, the synergy of immune enhancement and delivery properties remains a challenge. For instance, Inulin-derived nanoparticle adjuvants, such as AdvaxTM, have demonstrated the ability to enhance immune responses in vaccines targeting various viruses, including influenza and hepatitis B, but the poor antigen delivery effects and antigen protective ability limited the synergy effects of Inulin [23]. Moreover, the chitosan and alginate polymers have excellent properties for antigen protection and delivery ability but are not considered immunogenic against influenza whole virus [24, 25]. Overall, natural bio-polymers with synergy immune enhancement and antigen delivery effects remain few reported.

Existing approaches often require the use of coupling agents or activated connectors for antigen modification, which can introduce toxicities and affect the activity of the antigen. Moreover, subsequent purification processes can be inefficient and cumbersome [26]. Another critical concern is the biosafety of vaccine-related nanocarriers. It has been reported that typical cationic carriers like cationic liposomes, chitosan, and PEI can induce cell apoptosis [27]. For instance, charge-reversal functional gold nanoparticles prepared using a layer-by-layer technique have been employed to deliver small interfering RNA (siRNA) and plasmid DNA into cancer cells but exhibit some degree of toxicity towards cell proliferation [28]. Consequently, the development of non-toxic biomaterials with adjuvant activity as antigen delivery systems remains a significant bottleneck.

In this study, we discovered a natural amphiphilic exopolysaccharide named NAPS^*L.p*^ by screening the self-assembly properties of exopolysaccharides from 139 species of *Lactobacillus*. NAPS^*L.p*^ was utilized to encapsulate the model antigen OVA, resulting in the formation of nano-sized vaccines that were investigated for their immunostimulatory properties. Non-invasive bioimaging techniques were employed to visualize the pharmacokinetics following systemic administration, while the uptake of particles by dendritic cells was studied using confocal microscopy. The adjuvant capacity of NAPS^*L.p*^ was assessed by quantifying antibody production and T-cell activity after systemic administration. Moreover, the long-term immunity against B16-melanoma tumors was evaluated by challenging vaccinated mice with tumor cells. Furthermore, the effects of NAPS^*L.p*^ on influenza protection were examined, and NAPS^*L.p*^ exhibited stronger protective effects compared to the commonly used Al (OH)_3_ adjuvant. Above all, we systematically verified the superior adjuvant capacity and safety of using NAPS^*L.p*^ for improving vaccine design and performance.

## Results

### Purification and self-assembly characteristics of NAPS^*L.p*^

**Fig. 1:**
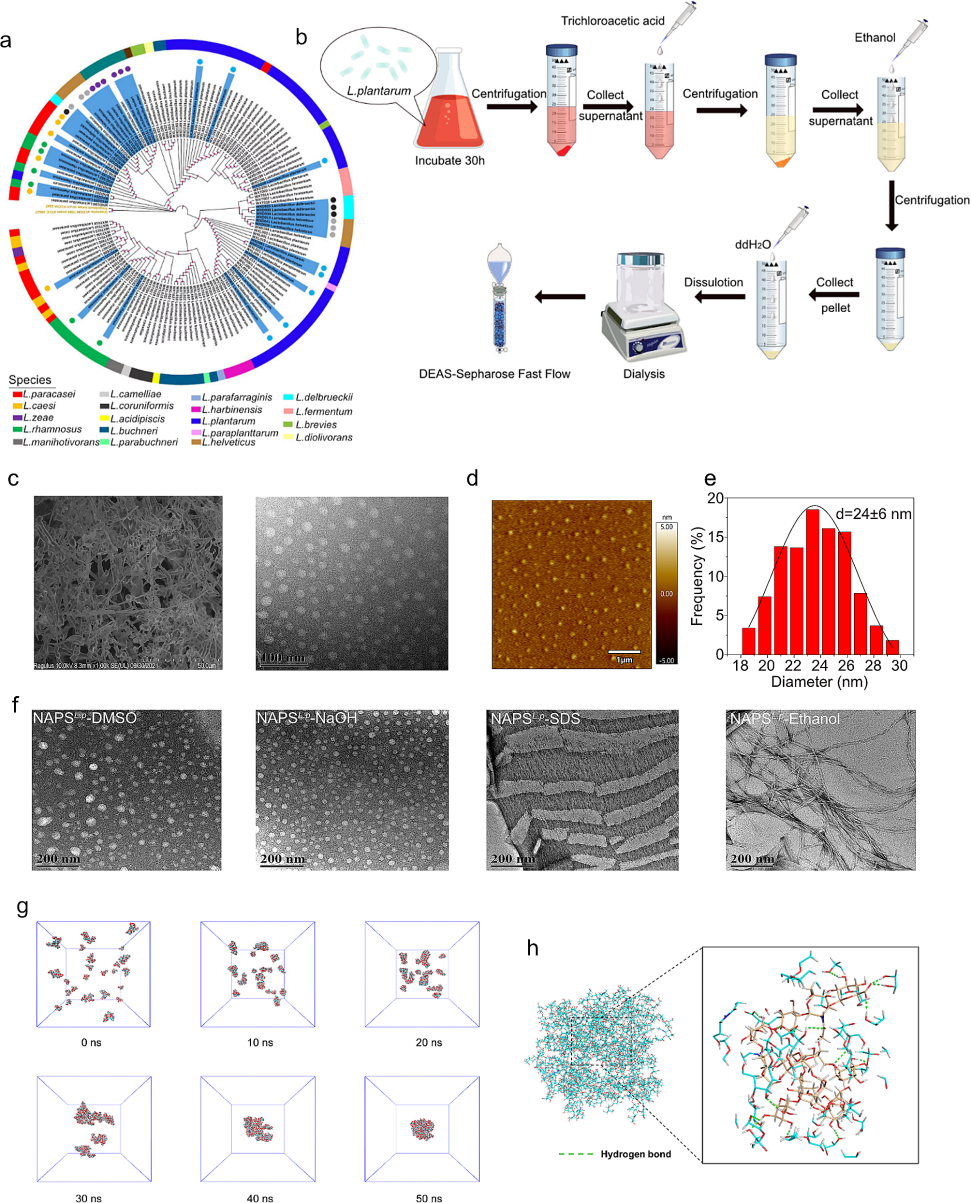
Screening, isolation, and self-assembly characterizations of NAPS^*L.p*^. (**a**) Neighbor join-based phylogenetic tree of the bacteria. (**b**) Generation and isolation of exopolysaccharide from selected bacteria. (**c**) SEM and TEM images of NAPS^*L.p*^. SEM image showed the fibrous shape of NAPS^*L.p*^ in water (left). The TEM image showed the morphology of NAPS^*L.p*^ after self-assembly inducing. (**d**) AFM image showed the three-dimensional morphology of self-assembled NAPS^*L.p*^. (**e**) The size of self-assembled NAPS^*L.p*^ about 24 ± 6 nm. (**f**) Morphology of self-assembled NAPS^*L.p*^ in different solutions. (**g**) Snapshots of the MD simulation systems at 0 ∼ 50 ns. (**h**) Schematic diagram of the interaction between a NAPS^*L.p*^ molecule

Natural exopolysaccharides are highly promising candidates for vaccine adjuvants due to their reported self-assembly properties and ability to enhance immunity. In this study, we collected a total of 139 wild bacterial species, including *Lactobacillus reuteri*, *Lactobacillus helveticus*, *Lactobacillus plantarum*, *Lactobacillus paracasei*, *Lactobacillus delburekii*, and *Lactobacillus rhamnosus*, to identify the best natural exopolysaccharides with self-adjuvant potential **(**Fig. 1a and Supplementary Table 1). The secretory exopolysaccharides were isolated using ethanol precipitation methods, followed by purification through a combination of ion-exchange chromatography and gel permeation chromatography. The resulting purified polysaccharide exhibited excellent homogeneity, as confirmed by the UV spectrum and high-performance gel permeation chromatography profiles (Supplementary Fig. 2 and Fig. 3**)**. To assess the self-assembly properties of the exopolysaccharides, we employed scanning electron microscopy (SEM), dynamic light scattering (DLS), Fourier-transform infrared spectroscopy (FTIR), and liquid chromatography-mass spectrometry (LC-MS) techniques (Supplementary Table 1, Tables 2, and Fig. 1**)**. Among the 139 bacterial species, exopolysaccharides derived from *Lactobacillus plantarum* (EPS-301) demonstrated strong self-assembly properties based on the SEM, FTIR, and DLS results (Supplementary Table 1, Tables 2, and Fig. 1**).** Further purification of the crude product was performed using DEAE Sepharose fast flow and Sepharose CL–6B gel column chromatography. The morphology of the polysaccharides, as observed under an electron microscope, appeared fibrous **(**Fig. 1c**)**. Following homogenization, the exopolysaccharides from *Lactobacillus plantarum* self-assembled into spherical nanoparticles with a size of approximately 30 nm at room temperature, exhibiting a bright and relatively homogeneous appearance in water (Fig. 1c and d, **and** Fig. 1e**).** Based on these findings, we designated the selected exopolysaccharides as natural self-assembly exopolysaccharides, naming them NAPS^*L.p*^.

**Fig. 2:**
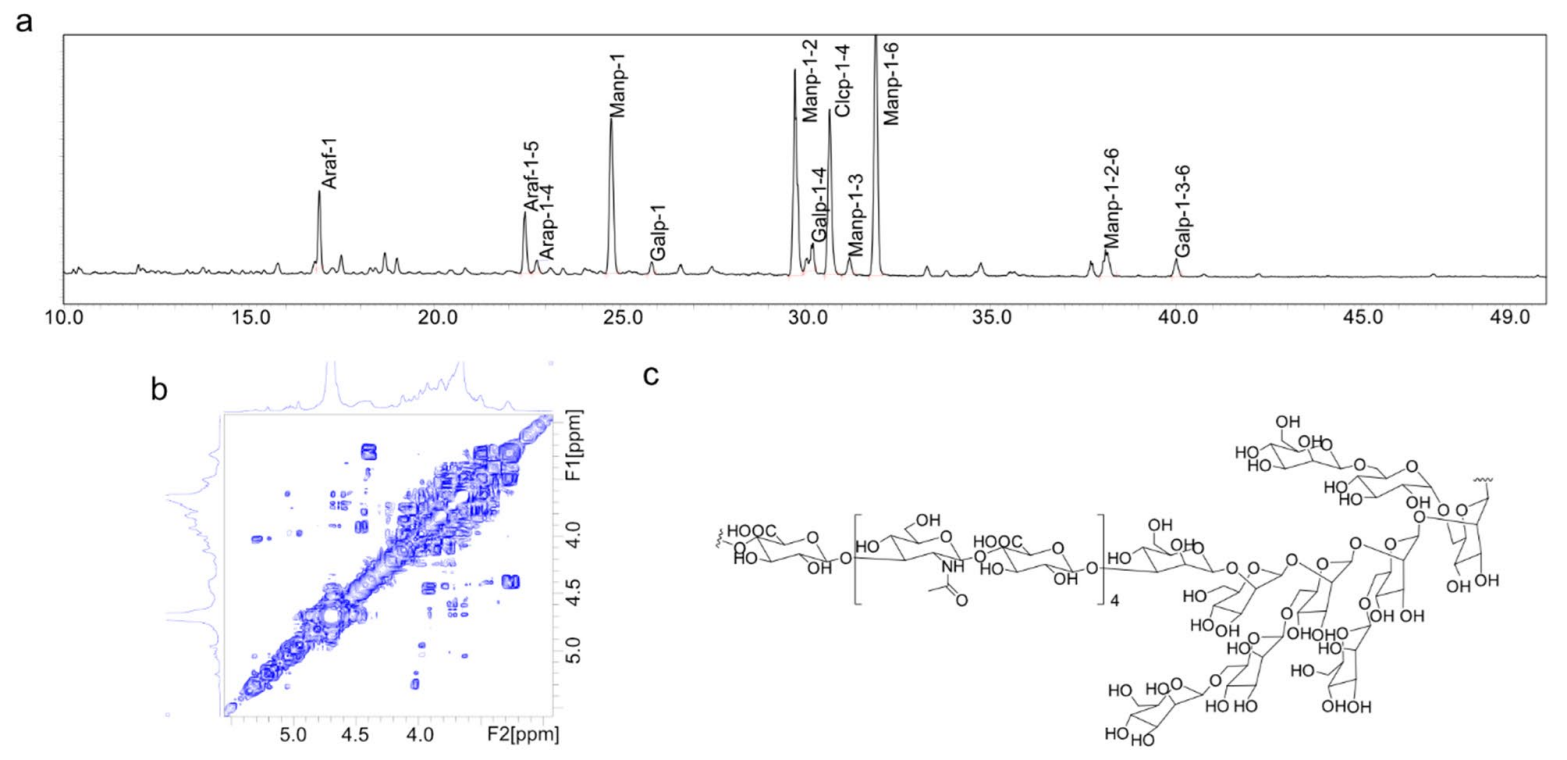
Chemical structure of NAPS^*L.p*^. (**a**) LC-MS results of NAPS^*L.p*^. (**b**) ^1^H-^1^H-COSY of NAPS^*L.p*^. (**c**) Chemical structure of NAP^*L.p*^ from results of LC-MS and NMR

To further understand the self-assembly behavior of NAPS^*L.p*^, we investigated its response to different solvents, including SDS (0.01 M), DMSO, NaOH (0.01 M), and 50% ethanol. The size of NAPS^*L.p*^ nanoparticles in DMSO were measured to be 26.42 ± 7 nm, which differed from that in water (Fig. 1f and Supplementary Fig. 4a). Similar morphologies were observed in NaOH solutions (Supplementary Fig. 4b), with a reduced size of approximately 24 ± 7 nm for NAPS^*L.p*^. When treated with SDS (0.01 M), the morphology of NAPS^*L.p*^ appeared as a network structure (Supplementary Fig. 4c). In 50% ethanol, the morphology of NAPS^*L.p*^ exhibited nanofiber structures (Supplementary Fig. 4d), indicating the gel formation of NAPS^*L.p*^ in 50% ethanol solution. These findings provide insights into the self-assembly behavior of NAPS^*L.p*^ in different solvents, enhancing our understanding of its properties.

To gain a more detailed understanding of the nanoscale self-assembly mechanism, molecular dynamics (MD) simulations were performed on NAPS^*L.p*^ molecules in water. Snapshots were captured at different time points to examine the changes in molecular configuration and self-assembly behavior during the simulation process. As depicted in Fig. 1g and Supplementary Fig. 5a, 30 NAPS^*L.p*^ molecules were initially randomly distributed in the solution at 0 ns. At 10 ns, some molecules began to aggregate into clusters. Finally, all 30 NAPS^*L.p*^ molecules clustered together, forming a stable spherical nanocluster at 50 ns. This observation confirmed the spontaneous aggregation behavior of NAPS^*L.p*^ molecules in aqueous solution.

Several interactive forces play a role in the self-assembly of nanoparticles, including hydrogen bonding, hydrophobic interactions, electrostatic interactions, and van der Waals forces. To investigate these forces, the number of hydrogen bonds formed between the 30 NAPS^*L.p*^ molecules was calculated throughout the simulation. As shown in Supplementary Fig. 5b, the number of intermolecular hydrogen bonds increased progressively over time, indicating a decrease in the distance between NAP^*L.p*^ molecules. Additionally, we analyzed the number of hydrogen bonds between NAPS^*L.p*^ molecules and water molecules during the simulation, and the results (Supplementary Fig. 5c) revealed a reduction in the hydrogen bonds between NAPS^*L.p*^ molecules and water molecules. This reduction indicated the tendency of NAPS^*L.p*^ molecules to self-assemble, with intermolecular hydrogen bonding being one of the primary forces driving molecule aggregation. Furthermore, Fig. 1h illustrates the analysis of the interaction between a NAPS^*L.p*^ molecule located in the center of a spherical nanocluster and the surrounding NAPS^*L.p*^ molecules. The figure demonstrates that NAPS^*L.p*^ molecules primarily self-assemble through the formation of numerous hydrogen bonds between the hydroxyl or carbonyl groups on the molecular chain and the surrounding NAPS^*L.p*^ molecules.

Hydrophobic interactions between water molecules and hydrophobic groups are another driving force for the self-assembly of nanoparticles. The degree of hydrophobicity can be approximated by the solvent-accessible surface area (SASA). In this study, we analyzed the SASA of the entire system during the simulation process to evaluate the compactness of NAPS^*L.p*^ molecules in the solution. As shown in Supplementary Fig. 5d, the SASA values of all polysaccharide molecules in the system decreased from 528.715 nm^2^ at 0 ns to 372.145 nm^2^ at the end of the 50 ns simulation. The system reached stability after 50 ns. The decrease in SASA indicated that the NAPS^*L.p*^ molecules became more compact, limiting their contact with water molecules. This finding suggests that NAPS^*L.p*^ molecules aggregate through hydrophobic interactions. The results shed light on the molecular mechanisms underlying the self-assembly process and enhance our understanding of the properties of NAPS^*L.p*^ as a potential self-adjuvant for immunity enhancement.

### Molecular structure and characteristics of NAPS^*L.p*^

We assessed the homogeneity and molecular weight of the polysaccharides. The average molecular weight was determined to be approximately 84.3 kDa by calculating the standard dextrans and measuring the elution time of the polysaccharides. The CD spectra of NAPS^*L.p*^(Supplementary Fig. 6) revealed that the secondary conformational structures of NAPS^*L.p*^ were primarily composed of α-helices and β-sheets. Analysis of the pure polysaccharide composition using high-performance liquid chromatography (HPLC) indicated the presence of galactosamine hydrochloride, arabinose, glucosamine hydrochloride, galactose, glucose, mannose, and glucuronic acid, with a molar ratio of 3.3:1:2.2:1:10.5:1:3.1 (Supplementary Fig. 7).

To gain further insights into the molecular structure of NAPS^*L.p*^, we conducted additional investigations using Fouriertransform infrared spectroscopy (FTIR) spectra, X-ray photoelectron spectroscopy (XPS), rheometer tests, and smallangle X-ray scattering (SAXS) analysis (Supplementary Figs. 8–11).

We defined the glycoside linkages of polysaccharides by gas chromatography-mass spectrometer (GC–MS) analysis **(**Fig. 2a and Supplementary Table 3). Twelve sugar residues of the polysaccharides could be elucidated as 2,3,5-Me_3_-Araf, 2,3-Me_2_-Araf, 2,3-Me_2_-Arap, 2,3,4,6-Me_4_-Manp, 2,3,4,6-Me_4_-Galp, 3,4,6-Me_3_-Manp, 2,3,6-Me_3_-Galp, 2,3,6-Me_3_-Glcp, 2,4,6-Me_3_-Manp, 2,3,4-Me_3_-Manp, 3,4-Me_2_-Manp, 2,4-Me_2_-Galp with the molar of 6.4:5.6:1.2:16.6:1:19.2:3.2:15.5:1.7:24.7:3.4:1.5, The corresponding link method is Araf-(1→, →5)-Araf-(1→, →4)-Arap- (1→, Manp-(1→, Galp-(1→, →2)-Manp-(1→, →4)-Galp-(1→, →4)-Glcp-(1→, →3)-Manp-(1→, →6)-Manp-(1→, →2,6)-Manp-(1→, →3,6)-Galp- (1→, respectively. Nuclear magnetic resonance (NMR), including ^1^H, DEPT 135, and 2D NMR spectra (^1^H-^1^HCOSY, HSQC, HMBC, and NOESY), was further carried out to reveal the NAPS^*L.p*^ structure (Fig. 2b and Supplementary Fig. 12), all glycosidic bond signals were speculated (Supplementary Table 4). Based on the results of glycosidic linkage analysis and NMR spectra analysis, the main chain connection of NAPS^*L.p*^ was →3)-β-D-Galp-(1→4)-β-D-GlcpNAc-(1→3)-Man-1→2-Man-1→2,6-Man-1→2,6-Man-1→ (Fig. 2c**).**

**Fig. 3:**
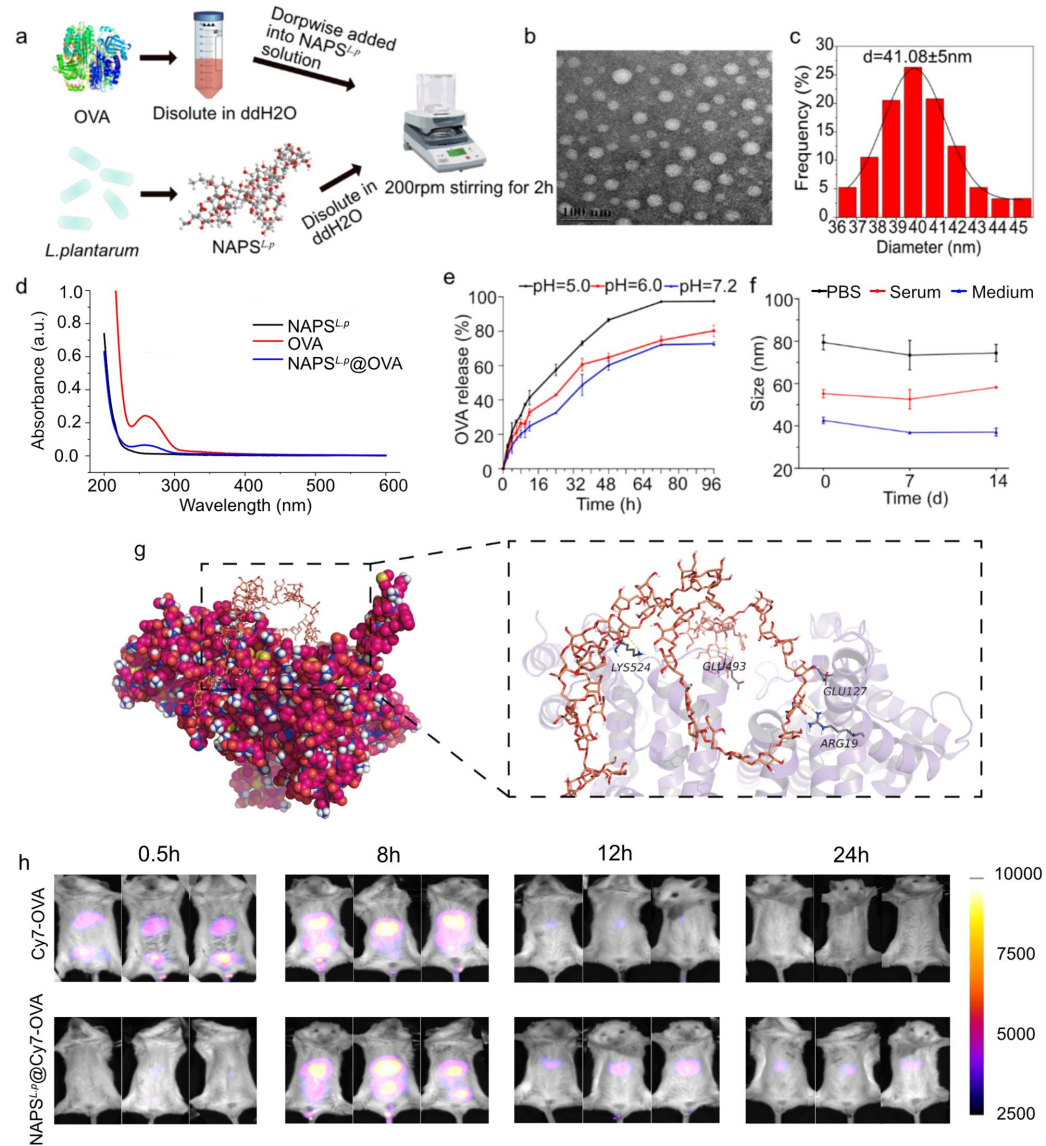
Characterizations of NAPS^*L.p*^ @OVA. (**a**) Preparation procedure of NAPS^*L.p*^@OVA. (**b**) TEM images of NAPS^*L.p*^@OVA. (**c**) Size distributions of NAPS^*L.p*^@OVA. (**d**) UV spectrum of NAPS^*L.p*^@OVA. (**e**) OVA releasing curve of NAPS^*L.p*^@OVA in different pH conditions. (**f**) The stability of NAPS^*L.p*^@OVA under various buffer conditions. **g**) The structure mode of NAPS^*L.p*^-OVA. OVA and NAPS^*L.p*^ are marked in purple and orange, respectively, and NAPS^*L.p*^ is depicted as a bar (C: orange; H: white; O: red; N: blue; hydrogen bonds: indicated by yellow dotted lines) The bond length is 2.10Å, 2.37Å, 2.43Å, and 2.68Å. (**h**) In vivo fluorescence imaging of Cy7-OVA and NAPS^*L.p*^@Cy7-OVA at timed intervals after intravenous injection. The data are presented as mean ± SD (*n* = 3)

### Characteristics of NAPS^*L.p*^@OVA nanoparticles

NAPS^*L.p*^ and OVA were mixed to create the NAPS^*L.p*^@OVA nanoparticles (Fig. 3a). These nanoparticles had a mean size of 41.32 ± 3.14 nm (PDI: 0.121 ± 0.04) for NAPS^*L.p*^ and 83.98 ± 1.62 nm (PDI: 0.142 ± 0.06) for NAPS^*L.p*^@OVA, as determined by DLS (Supplementary Table 5). The TEM image showed that NAPS^*L.p*^@OVA had a uniform spherical shape **(**Fig. 3b and Fig. 3c). The successful encapsulation of OVA was confirmed by UV-Vis and FTIR spectroscopy. CD spectroscopy revealed the structure of OVA in NAPS^*L.p*^@OVA remained intact (Fig. 3d, Supplementary Fig. 13 and Fig. 14). The contact angle analysis indicated that both NAPS^*L.p*^ and NAPS^*L.p*^@OVA exhibited hydrophilicity, enhancing their bioavailability (Supplementary Fig. 15).

NAPS^*L.p*^@OVA had an antigen loading capacity of 57.98 ± 1.65% and an encapsulation efficiency of 87.30 ± 2.64% (Supplementary Table 5). Release experiments showed that less than 25% of OVA was released within 24 h at pH 7.2, while at pH 6.0 and 5.5, more than 40% and 60% of OVA were released, respectively **(**Fig. 3e**)**. This pH-responsive release indicated that potential for targeted antigen delivery and immune responses [29]. The stability of NAPS^*L.p*^@OVA in various solvents, including PBS, serum, and cell medium, was confirmed for 14 days (Fig. 3f). The assembly between NAPS^*L.p*^ and OVA protein was verified using stoichiometric calculation and molecular docking analysis (Fig. 3g). Four hydrogen bonds were observed between the active site of OVA protein and NAPS^*L.p*^ segments. The calculated dock score value for NAPS^*L.p*^@OVA protein was − 7.505 kcal/mol. To assess antigen retention, Cy7-labeled OVA was injected into mice. The fluorescent signal of NAPS^*L.p*^@Cy7-OVA was detectable even after 24 h, indicating prolonged antigen retention and stimulation capability for antigen-presenting cells (APCs) (Fig. 3h).

### Immunity activation and antigen presentation mediated by NAPS^*L.p*^@OVA

**Fig. 4:**
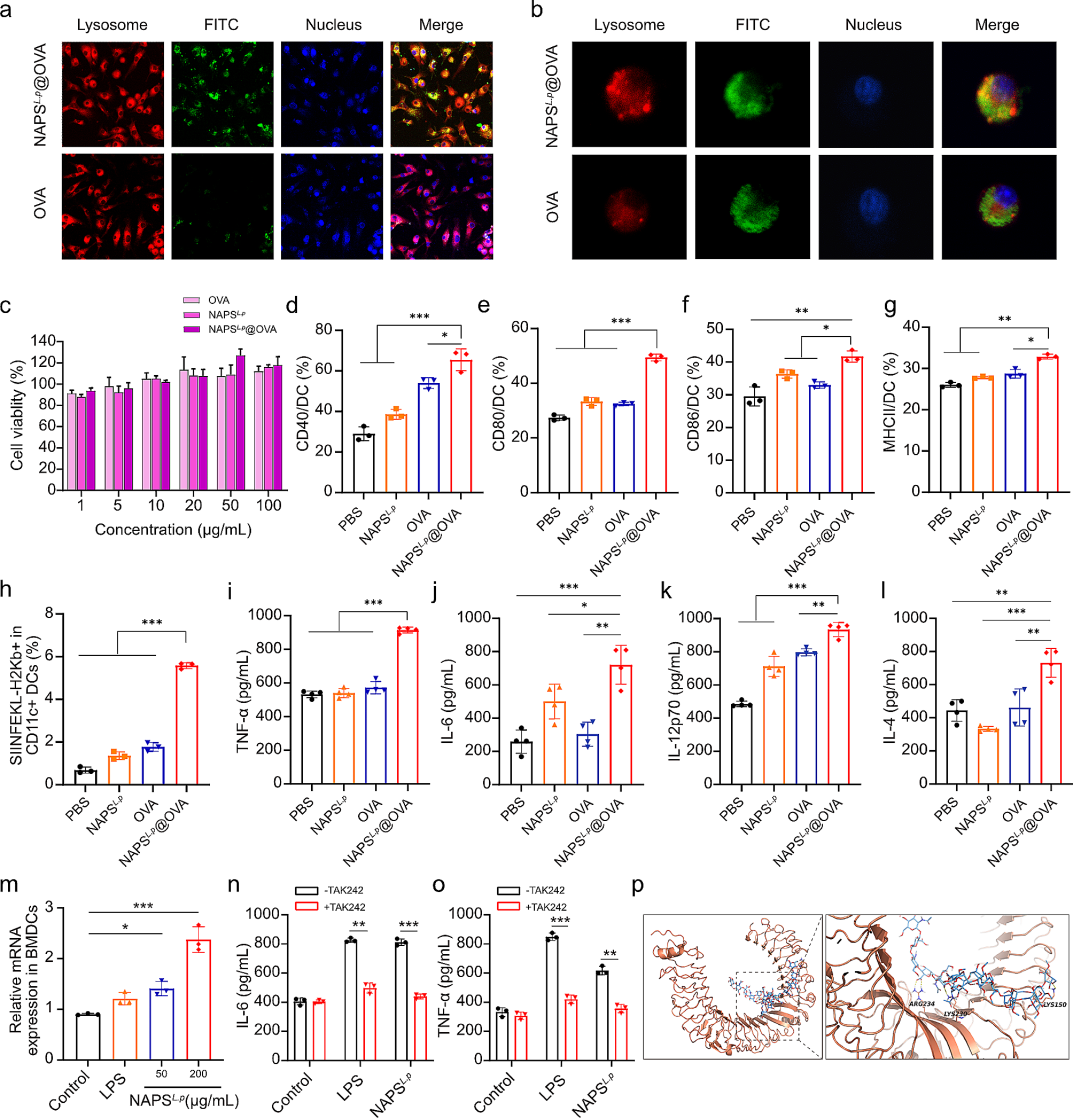
Immunity activation and antigen presentation mediated by NAPS^*L.p*^@OVA. (**a**) Confocal images of primary dendritic cells co-incubating with NAPS^*L.p*^@OVA-FITC or FITC-OVA. (**b**) High-solution images of primary dendritic cells. These results indicated that the FITC-OVA exhibited stronger internalization abilities after NAPS^*L.p*^ encapsulation. (**c**) Cytotoxicity of BMDCs after coculturing for 24 h with NAPS^*L.p*^, OVA and NAPS^*L.p*^@OVA. The expression levels of (**d**) CD40, (**e**) CD80, (**f**) CD86, and (**g**) MHC-II, were measured with flow cytometers. (**h**) The percentage of SIINFEKL-H-2Kb^+^ on BMDCs treated with NAPS^*L.p*^, OVA, and NAPS^*L.p*^@OVA. The cytokine secretion levels of (**i**) TNF-α, (**j**) IL-6, (**k**) IL-12p70, and (**l**) IL-4 were measured with ELISA assays. (**m**) The mRNA transcription levels of TLR4 in DCs cells. IL-6 (**n**) and TNF-α (**o**) in the supernatant of DCs were tested by ELISA kit. (**p**) Molecular docking of NAPS^*L.p*^ and TLR4. The data are presented as mean ± SD (*n* = 3). One-way ANOVA with a Tukey multiple comparisons test was used for statistical analysis. *, *p* < 0.05; **, *p* < 0.01; ***, *p* < 0.001

Dendritic cells (DCs) were tested for antigen uptake using FITC-labeled OVA (OVA-FITC) or NAPS^*L.p*^-bound OVA-FITC (NAPS^*L.p*^@OVA-FITC). Results showed that NAPS^*L.p*^@OVA-FITC had higher intracellular uptake in BMDCs compared to OVA-FITC alone. Co-localization of NAPS^*L.p*^@OVA-FITC with lysosomes in primary dendritic cells was observed (Fig. 4a, and 4b). A hemolysis test evaluated the hemolysis of NAPS^*L.p*^@OVA to red blood cells, and the results showed that no significant hemolysis occurred (Supplementary Fig. 16), which indicated good biocompatibility. Cytotoxicity tests on BMDCs and RAW264.7 showed no significant toxicity even at high concentrations (100 µg/mL) of NAPS^*L.p*^@OVA (Fig. 4c and Supplementary Fig. 17). NAPS^*L.p*^@OVA increased the expression of CD40, CD80, CD86, and MHC II on BMDCs, indicating DC maturation and activation (Fig. 4d-g and Supplementary Fig. 18). As indicated in Fig. 4h and supplementary Fig. 19, we further observed that the treatments of NAPS^*L.p*^@OVA increased the expression level of SIINFEKL-H-2K^b^ complexes on the surface of BMDCs by 2.87-fold compared to the “OVA” group, suggesting the NAPS^*L.p*^@OVA nanovaccines efficiently enhance the efficacy of antigen cross-presentation of DCs. The levels of TNF-α, IL-6, IL-12p70, and IL-4 cytokines were higher in the cultural supernatant of BMDCs treated with NAPS^*L.p*^@OVA compared to control groups, suggesting cellular and humoral immune response activation (Fig. 4i-l). To investigate the signaling pathway mediated by NAPS^*L.p*^, the mRNA transcript levels of TLR4 in DC cells were measured by RT-PCR after exposure of DC cells to various concentrations of NAPS^*L.p*^. The results showed that NAPS^*L.p*^ upregulated TLR4 expression in the DCs cells compared to the control group (Fig. 4m). To confirm further that NAPS^*L.p*^ exerts its effects through the TLR4 signaling pathway, we used the TLR4 antagonist TAK-242 to determine whether the NAPS^*L.p*^-induced secretion of IL-6 and TNF-α was due to the activation of specific TLR4. As shown in Fig. 4n and o, the least number of cytokines was produced when DC cells were exposed to the medium alone. However, the secretion of TNF-α and IL-6 was significantly inhibited in DC cells pre-incubated with TAK-242 and then added to NAPS^*L.p*^ compared to the NAPS^*L.p*^ treatment alone, which further demonstrated that the NAPS^*L.p*^ activates the TLR4 pathway in BMDC. In addition, Simulated calculation suggested that NAPS^*L.p*^ binds to TLR4 protein mainly through hydrogen bonds and hydrophobic forces (Fig. 4p). After binding to NAPS^*L.p*^, the conformation of the loop region of TLR4 protein will change to a certain extent, thereby activating downstream signaling pathways and exerting its adjuvant activity. Mice were subcutaneously immunized with NAPS^*L.p*^@OVA to evaluate humoral immune responses. We specifically analyzed antibodies targeting individual components of the vaccine, focusing on IgG, IgG1, and IgG2b. Notably, both NAPS^*L.p*^@OVA and aluminum OVA groups exhibited higher titers of IgG, IgG1, and IgG2b compared to the OVA-treated group (Supplementary Fig. 20), indicating that NAPS^*L.p*^ encapsulation enhances antibody production and acts as an immune adjuvant. To assess potential in vivo toxicities of NAPS^*L.p*^@OVA, we performed hematoxylin and eosin (H&E) staining after a single administration. One week post-administration, mice were sacrificed, and liver, spleen, heart, and lung samples were collected. The results demonstrated that NAPS^*L.p*^@OVA did not induce pathological changes (Supplementary Fig. 21), confirming the low toxicity profile of NAPS^*L.p*^.

### Antitumor effect of the nanovaccines in a subcutaneous tumor model

**Fig. 5:**
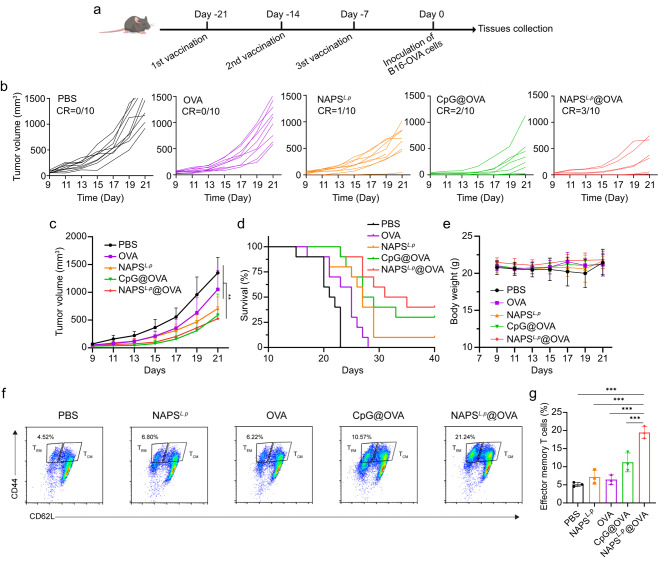
Anti-tumor efficacy of prophylactic immunization in a subcutaneous B16-OVA melanoma model. (**a**) Schematic illustration of prophylactic immunization for subcutaneous B16-OVA melanoma model. (**b**) Individual tumor growth curves for mice after different treatments. (**c**) Growth curves of the average volumes in the indicated groups (*n* = 10). (**d**) Survival curves of mice with different treatments as indicated (*n* = 10). (**e**) Body weight changes of mice in different groups (*n* = 10). (**f**) Representative scatter plots and gating information derived from analysis of effector memory T cells (T_EM_, CD3^+^ CD8^+^ CD44^+^ CD62L^−^) cells in Spleen. (**g**) Quantitative analysis of T_EM_ cells in Spleen (*n* = 3). Data are shown as mean ± SD. One-way ANOVA with a Tukey multiple comparisons test was used for statistical analysis. **, *p* < 0.01, ***, *p* < 0.001

Mice were vaccinated with B16-OVA melanoma tumor cells to study anticancer immunity. The mice received subcutaneous immunizations of NAPS^*L.p*^, OVA_257 − 264_, CpG@OVA_257 − 264_, NAPS^*L.p*^@OVA_257 − 264_ and PBS three times at one-week intervals **(**Fig. 5a**)**. After vaccination, the mice were challenged with B16-OVA melanoma cells. The group treated with CpG@OVA showed inhibited tumor progression compared to the control group injected with PBS. Notably, the NAPS^*L.p*^@OVA-treated group exhibited the best therapeutic effect, with significantly prolonged animal survival and complete tumor elimination in three mice **(**Fig. 5b, c, d**)**. In the CpG@OVA group, 2 out of 10 mice showed complete tumor regression. In comparison, only 1 out of 10 mice in the NAPS^*L.p*^ group exhibited regression, and no complete regression was observed in the other groups **(**Fig. 5b**)**. Importantly, the body weights of the vaccinated mice slightly increased, indicating minimal effects on their weight **(**Fig. 5e**)**. Given the crucial role of immune memory in long-term antitumor benefits, therefore, we investigated the persistence of immune memory in vivo after vaccination with NAPS^*L.p*^@OVA_257 − 264_. As indicated in Fig. 5f and g, we investigated the immune memory T cells in the spleens on day 60 since the fast immunization by flow cytometry. It is worth noting the proportions of T_EM_ (CD3^+^ CD8^+^ CD44^+^ CD62L^−^) and T_CM_ (CD3^+^ CD8^+^ CD44^+^ CD62L^+^) in the mice immunized with NAPS^*L.p*^@OVA reveals a 4.70-fold and 2.10-fold increase, compared with that in the control group (Fig. 5f, g and Supplementary Fig. 22). Furthermore, NAPS^*L.p*^@OVA vaccination induced the greatest increase in tissue resident memory T cells compared with those in the other groups (Supplementary Fig. 23). These results demonstrated that the NAPS^*L.p*^@OVA nanovaccines can generate sustained antigen-specific immune memory effect for preventing antigen-specific tumor occurrence.

**Fig. 6:**
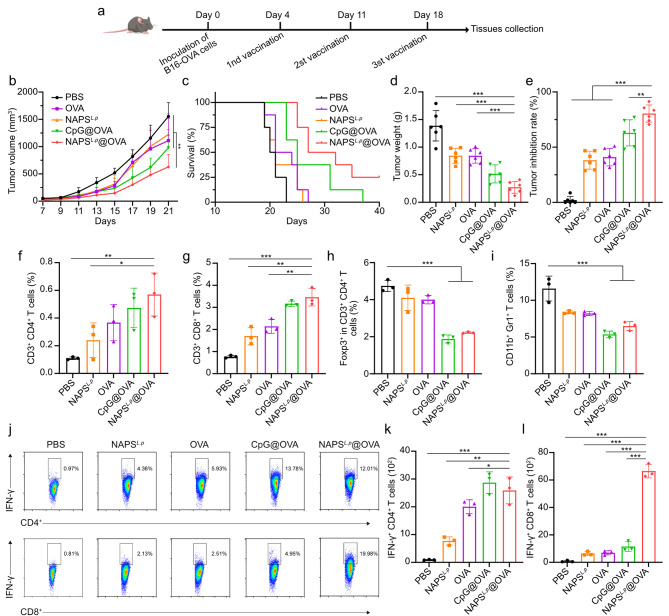
Anti-tumor efficacy in a B16-OVA melanoma tumor model. (**a**) Schematic illustration of therapeutic immunization for subcutaneous B16-OVA melanoma model. (**b**) Growth curves of the average volumes in the indicated groups (*n* = 8). (**c**) Survival curves of mice with different treatments as indicated (*n* = 8). (**d**) The tumor weight of excised B16-OVA tumors from mice in each group (*n* = 6). (**e**) Tumor inhibition rates are calculated according to the tumor weight (*n* = 6). (**f**) Quantitative analysis of CD3^+^ CD4^+^ T cells. (**g**) CD3^+^CD8^+^ T cells. (**h**) CD3^+^ CD4^+^ Foxp3^+^ Tregs, and (**i**) MDSCs (CD11b^+^ Gr-1^+^). (**j**) Infiltrating the tumor tissues, as examined by flow cytometry (*n* = 3). Flow cytometric analysis assessed IFN-γ^+^ CD4^+^ T (**k**) cells and CD8^+^ T (**l**) cells in the tumor tissues of mice in the B16-F10 tumor model. Data are shown as mean ± SD. One-way ANOVA with a Tukey multiple comparisons test was used for statistical analysis. *, *p* < 0.05; **, *p* < 0.01; ***, *p* < 0.001

The efficacy of NAPS^*L.p*^@OVA_257 − 264_ was evaluated in mice with B16-OVA melanoma. Mice were divided into five groups and received different treatments **(**Fig. 6a**)**. The tumor inhibition rates were 2.04%, 38.25%, 40.93%, 62.95%, and 80.45% for PBS, NAPS^*L.p*^, OVA_257 − 264_, CpG@OVA_257 − 264_, and NAPS^*L.p*^@OVA_257 − 264_ groups, respectively **(**Fig. 6e**)**. NAPS^*L.p*^@OVA showed the highest inhibition, but all vaccine formulations inhibited tumor growth (Supplementary Fig. 24 and Fig. 6b, d**)**. NAPS^*L.p*^@OVA also prolonged animal survival **(**Fig. 6c**)** without causing weight loss (Supplementary Fig. 25).

Tumor-infiltrating immune cells were analyzed, and the NAPS^*L.p*^@OVA groups showed significantly increased CD3^+^CD4^+^ and CD3^+^CD8^+^ T cell infiltration in tumor tissues compared to the control group **(**Fig. 6f, g and Supplementary Figs. 26, 27). NAPS^*L.p*^@OVA vaccination effectively reduced regulatory T cells (Tregs, CD3^+^CD4^+^ Foxp3^+^ T cells) and myeloid-derived suppressor cells (MDSCs, CD11b^+^Gr-1^+^) in tumor tissues **(**Fig. 6h, i and Supplementary Figs. 28, 29). Additionally, NAPS^*L.p*^@OVA induced a significant increase in tumor-specific interferon-γ (IFN-γ) secreting CD4^+^ and CD8^+^ T cells **(**Fig. 6j, k, l**)**. NAPS^*L.p*^@OVA treatment also increased CD3^+^ CD4^+^ and CD3^+^ CD8^+^ T cells in the spleen (Supplementary Figs. 30, 31). Tumor tissue histology via H&E staining reveals that among all groups, NAPS^*L.p*^@OVA exhibits the lowest cell density, the largest intercellular space, and the most pronounced nucleus atrophy (Supplementary Fig. 32a). Immunofluorescence assays for CD4 and CD8 markers were conducted. Supplementary Fig. 32b demonstrates that the expressions of CD4 and CD8 are notably elevated in tumor tissues of mice treated with NAPS^*L.p*^@OVA, compared to control groups, indicating heightened infiltration of CTLs in tumors prompted by NAPS^*L.p*^@OVA. These findings demonstrate that NAPS^*L.p*^@OVA effectively activates local and systemic antigen-specific immune responses. The study highlights the potential of NAPS^*L.p*^ as a prophylactic and therapeutic vaccine for solid tumors.

### NAPS^*L.p*^-based vaccination offers strong influenza protection

**Fig. 7:**
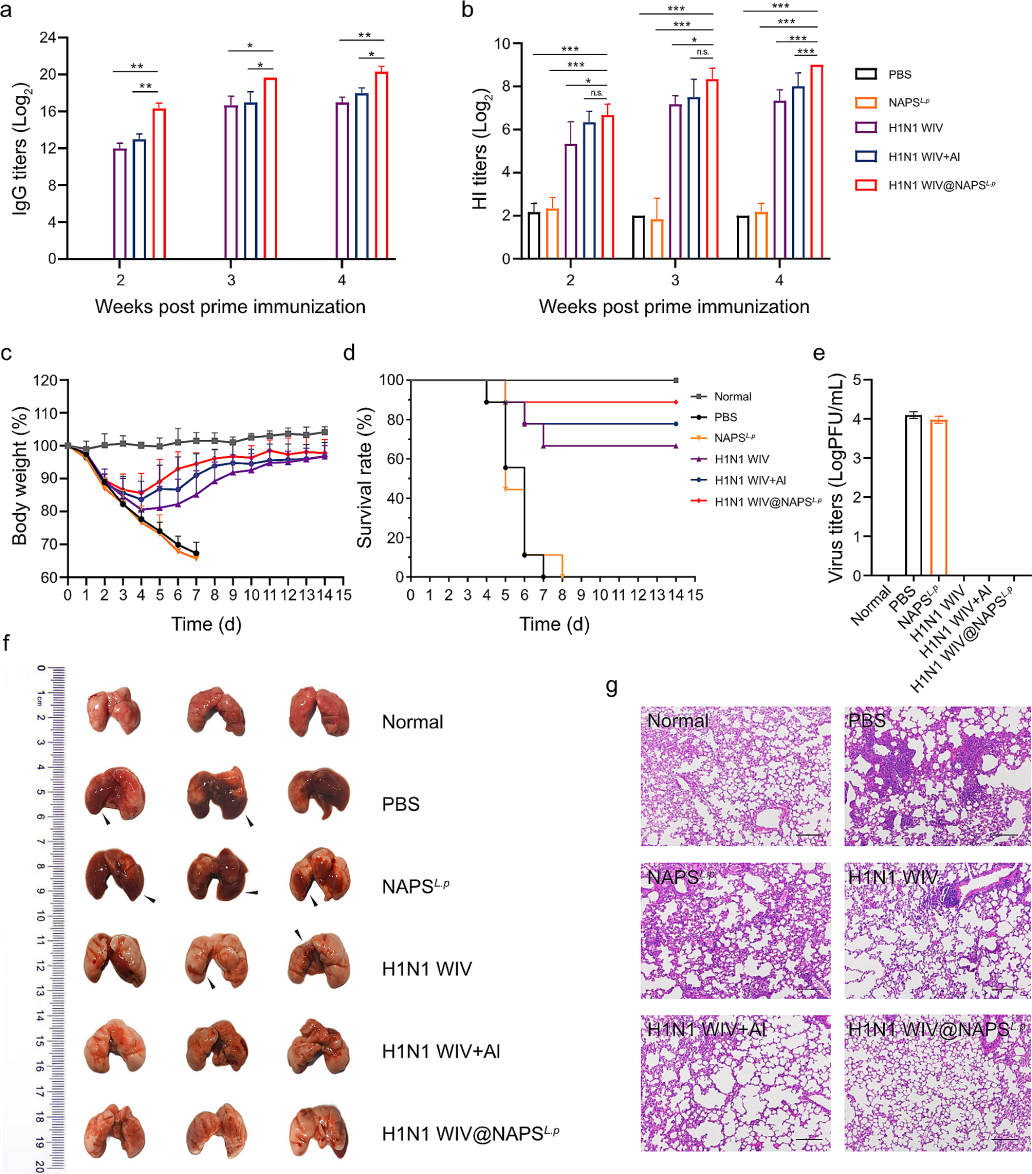
Immunization with NAPS^*L.p*^-based influenza vaccine protects mice against H1N1 influenza virus infection. (**a**) H1N1 WIV-specific serum IgG titers were determined by indirect ELISA. (**b**) Serum hemagglutination inhibition (HI) levels were detected by HI assay using 4 hemagglutinating units of the influenza virus strain. (**c**) Body weight changes in the vaccinated mice were challenged with 10^6^ PFU of H1N1 influenza virus 28 days post-primary immunization. (**d**) Change in the survival of vaccinated mice was challenged with 10^6^ PFU of H1N1 influenza virus 28 days after initial immunization. Mice who lost more than 25% of their body weight were euthanized according to guidelines. *n* = 9. (**e**) Virus load in the lung (*n* = 3) on day 5 p. i. (**f**) The pathological changes in lungs (*n* = 3) on day 5 p. i. (**g**) Representative histopathological changes in H&E-stained lung tissues (*n* = 3) on day 5 p.i. magnification 200×. Data are shown as mean ± SD. One-way ANOVA with a Tukey multiple comparisons test was used for statistical analysis. *, *p* < 0.05; **, *p* < 0.01; ***, *p* < 0.001

To test the effectiveness of NAPS^*L.p*^ in response to H1N1 WIV, mice were immunized subcutaneously with H1N1 WIV alone, NAPS^*L.p*^ alone, H1N1 WIV plus Al(OH)_3_, H1N1 WIV, and NAPS^*L.p*^ complexes for two doses. Serum samples were collected on days 14, 21, and 28 after the first immunization to measure IgG and hemagglutination-inhibiting (HI) antibody titers **(**Fig. 7a and  b**)**. The H1N1 WIV@NAPS^*L.p*^ group showed significantly higher IgG and HI titers compared to the H1N1 WIV group on all three-time points (*p* < 0.5 or *p* < 0.01).

To assess the protective efficacy of the H1N1 WIV@NAPS^*L.p*^ vaccine, mice were intranasally challenged with the H1N1(A/PR/8) virus on day 28 after the initial immunization. Body weight changes and survival rates were monitored for two weeks post-challenge **(**Fig. 7c and d**)**. Mice immunized with H1N1 WIV@NAPS^*L.p*^ showed recovery after a slight weight loss, and the survival rate in this group was 88.8%, demonstrating better protection compared to the H1N1 WIV plus Al (OH)_3_ group. None of the mice in the H1N1 WIV@NAPS^*L.p*^ group had detectable virus titers **(**Fig. 7e**)**. In contrast, mice immunized with H1N1 WIV alone experienced severe weight loss and some died. Lung pathology analysis revealed severe damage in the lungs of mice in the PBS group on day 5 post-infection, including swelling, hyperemia, and hemorrhage. In contrast, mice immunized with H1N1 WIV, H1N1 WIV plus Al (OH)_3_, or H1N1 WIV@NAPS^*L.p*^ showed only slight pathological changes **(**Fig. 7f, g and Supplementary Fig. 33). These results indicate that vaccination with NAPS^*L.p*^-based H1N1 inactivated vaccine protected mice from a lethal influenza challenge.

## Discussion

In our study, we discovered a natural polysaccharide with self-assembly properties among 139 bacterial species. Surprisingly, most of these polysaccharides exhibited self-assembly properties and acted as immune adjuvants. These polysaccharides could adopt various nanoscale conformations in solution, such as spherical nanoparticles and nanofibers, which endowed them with multiple functions [30, 31]. The diverse self-assembly properties observed in these polysaccharides can be attributed to variations in molecular weight, residue compositions, and solubility [32]. Based on our SEM, FTIR, and DLS findings, we identified a novel natural polysaccharide from *L.plantarum* with remarkable self-assembly properties. This polysaccharide was named NAPS^*L.p*^. Analysis using HPGPC revealed that NAPS^*L.p*^ is composed of galactosamine hydrochloride, arabinose, glucosamine hydrochloride, galactose, glucose, mannose, and glucuronic acid. The distinct physical and chemical properties of these carbohydrates contribute to the exceptional self-assembly capabilities of NAPS^*L.p*^. As a result, we believe that NAPS^*L.p*^ exhibits stronger self-assembly abilities compared to other exopolysaccharides, such as those derived from fungus [33], *Klebsielsp* EPS of PHRC1.001 [34] and others [35, 36]. NAPS^*L.p*^ has abundant hydroxyl residues, allowing it to interact with protein antigens through hydrophobic interactions, hydrogen bonding, and electrostatic associations, driving assembly. Research has indicated that the notable immunoreactivity of EPS can be attributed to its chemical constituents, encompassing hydroxyl, carbon radical, and sulfate groups [37, 38]. The strong adjuvant activity of NAPS^*L.p*^ is attributed to its abundant mannose groups, which readily engage immune receptors [39, 40]. NAPS^*L.p*^ enhances immune responses by elevating specific serum IgG, IgG1, and IgG2a, which in turn enhances Th1/Th2 response [41]. Moreover, NAPS^*L.p*^ induces the functional maturation and cross-presentation of DCs by upregulating the expression of molecular markers on DCs surfaces, including CD40, CD80, CD86, and MHC-II, as well as OVA_257 − 264_ peptide (SIINFEKL)-MHCI. Mature DCs secrete TNF-α, IL-6, and IL-12p70, which enhances Th1 immunity [42]. In contrast, the expression of IL-4 activates the Th2 immune response [43]. Further studies confirmed that NAPS^*L.p*^ could trigger DC activation and antigen presentation by TLR4 in vitro. However, whether there are other pathways for NAPS^*L.p*^ still needs further verification. These findings reveal a novel mechanism related to the immune response of NAPS^*L.p*^ and confirm its potential as an adjuvant for vaccines.

Natural polysaccharides are widespread in organisms from prokaryotes to eukaryotes, serving as critical components involved in various life activities [44, 45]. They have been used for decades as building blocks to create functional biomaterials and can be modified to formulate derivatives for applications in delivery carriers [46, 47]. Additionally, they play essential roles in immune system function and immune response stimulation [48]. Natural polysaccharides possess favorable properties such as biocompatibility, renewability, non-toxicity, biodegradability, and low immunogenicity, making them promising adjuvant candidates [49]. However, obtaining immunopotentiators from natural sources with sufficient quantity, purity, and homogeneity is often challenging [50]. The NAPS^*L.p*^ that we reported demonstrates high quantity, purity, and homogeneity. It self-assembles into spherical nanoparticles of approximately 30 nm in size, exhibiting brightness and relatively uniform distribution in water. Therefore, we believe NAPS^*L.p*^ holds promise as a delivery platform.

Recent studies have reported the vaccine adjuvant effects of natural polysaccharides [42, 51]. However, the effectiveness of these polysaccharides as adjuvants is not satisfactory. For instance, the magnitude of RBD-specific antibody responses induced by PRBS-RBD protein nanovaccine was comparable to that of traditional RBD, only around 1.1-fold stronger than the traditional RBD protein vaccine [52]. In contrast, our study demonstrated that NAPS^*L.p*^, when used as an immune adjuvant to treat B16-OVA melanoma, exhibited more than a 3.8-fold increase in antibody levels compared to the untreated group, surpassing the performance of Al (OH)_3_. Additionally, current research efforts are focused on developing adjuvants that can enhance CD8^+^ T-cell responses and tailored antibody isotypes to improve vaccine responses. Many studies are exploring effective adjuvants and vaccine delivery systems for cancer vaccines [53, 54]. Our nanovaccine elicits immune responses from both CD4^+^ and CD8^+^ T cells and demonstrates excellent preventive effects against melanoma. Furthermore, NAPS^*L.p*^-based vaccination showed stronger protective effects against influenza compared to Al (OH)_3_ adjuvant.

Our study provides experimental evidence for a new co-assembly polysaccharide with self-assembly properties, optimal antigen packing and presentation, and the ability to induce a persistent anti-tumor immune response, all without the need for crosslinkers or chemical additives. In recent years, there has been a focus on finding safe and effective vaccine adjuvants and drug delivery systems for formulating mucosal vaccines. Natural polysaccharides, with their biocompatibility, biodegradability, non-toxicity, immunological properties, receptor recognition ability for antigen-presenting cells, and cytokine stimulation, hold great potential in this regard. Therefore, natural polysaccharide nanomaterials offer promising prospects for the development of mucosal vaccine adjuvants and delivery systems.

### Electronic supplementary material

Below is the link to the electronic supplementary material.

Supplementary Material 1

## Data Availability

No datasets were generated or analysed during the current study.

## Declarations

### Authors’ information

Shouxin Sheng, Haochi Zhang, and Xinyu Li equally contribute to this work as first authors.
