# Supplementary material for: Probiotic-derived amphiphilic exopolysaccharide self-assembling adjuvant delivery platform for enhancing immune responses
Source: J Nanobiotechnology. 2024 May 19;22:267. doi: 10.1186/s12951-024-02528-y (PMC11103965; doi:10.1186/s12951-024-02528-y)
Supplement: Supplementary file 1 — Supplementary Material 1 [file 12951_2024_2528_MOESM1_ESM.docx]

# Supplementary information

**Probiotic-Derived Amphiphilic Exopolysaccharide Self-Assembling Adjuvant Delivery Platform for Enhancing Immune Responses**

*Shouxin Sheng^1#^, Haochi Zhang^1#^, Xinyu Li^1#^, Jian Chen^1,2^, Pu Wang^1^, Yanchen Liang^1^, Chunhe Li^1^, Haotian Li^1^, Na Pan^1^, Xuemei Bao^1^,* *Mengnan Liu^1^*, *Lixia Zhao^2^, Xiaoyan Li^2^, Pingyuan Guan^2^, and Xiao Wang^1*^*

*^1^State Key Laboratory of Reproductive Regulation & Breeding of Grassland Livestock, School of Life Science, Inner Mongolia University, Hohhot, P.R. China 010021*

*^2^JinYuBaoLing Biopharmaceutical Co. Ltd., Inner Mongolia, Hohhot, P.R. China 010000*

**: To whom correspondence should be addressed.*

*#: These authors contributed equally to this work.*

## Experimental Section

1. **Materials and animals**

Ovalbumin (OVA) and OVA-fluorescein isothiocyanate (FITC) were plasmids by Sigma-Aldrich (USA) and Bersee (China). The thiol-modified oligodeoxynucleotide CpG (5′-tccatgacgttcctgacgtt-3′) was obtained from Synbio Technologies Co., Ltd (Suzhou, China). OVA_257-264_ (SIINFEKL) peptides were a customized product provided by GenScript Biotechnology Co., Ltd (Nanjing, China). Aluminum hydroxide adjuvant was purchased from Sigma-Aldrich (USA). The BCA protein assay kit was purchased from Solarbio (Beijing, China), and CCK-8 cell proliferation was purchased from Biosharp (BS350B, China). FluoLyso-^TM^ Red was purchased from Baisai (China), DAPI Staining Solution was purchased from Bioss (China), Near-Infrared Cy7 NHS ester (Excitation: 730 nm, Emission: 790 nm) was purchased from QiYue Technology (Xi’an, China). RPMI-1640 medium and heat-inactivated fetal bovine serum (FBS) were purchased from Thermo Fisher Scientific.

The specific-pathogen-free (SPF) female C57BL/6J mice were obtained from Beijing Speifu Biotechnology Co., Ltd. (Beijing, China), and fed under SPF conditions. All of the animal-related experimental protocols applied in this study were conducted under the standards of the Ethics Committee of Inner Mongolia University (SYXK2020-0006).

1. **Bacterial strains and Evolutionary tree analysis**

The 139 strains of lactic acid bacteria (LAB) were isolated from traditional dairy products. In addition, they were identified by biochemical analysis and by sequence analysis of the 16S rRNA genes. The phylogenetic relationships, based on conserved genes of the 139 Lactic acid bacteria family strains were analyzed using MEGA.10.0.

1. **Production and Purification of Polysaccharides**

Polysaccharides are obtained from lactic acid bacteria fermentation broth. Briefly, 1 mL of an overnight bacterial culture was inoculated into 50 mL of DeMan, Rogosa, and Sharpe (MRS) broth. After a 24-hour incubation at 37℃, bacterial cells were collected by centrifugation at 5,000 rpm for 15 min at 4℃. Further, it removes the bacterial pellet and collects the supernatants. Ethanol was added into the supernatants at 80% (w/v%) final concentration and allowed to precipitate at 4℃ for 12 h. The mixture solutions were centrifugated at 5000 rpm for 15 min and collected in the pellet. Dialyze the pellet using 8000-12000 Da membrane for 24 h and lyophilized to obtain the crude polysaccharide.

The crude polysaccharide was dissolved in distilled water to a concentration of 10 mg/mL and further purified by DEAE Sepharose Fast Flow (GE, USA) column (D 2.6 × 40 cm). The eluted solution contained polysaccharides that were collected, dialyzed against distilled water at 4℃ for two days, and then lyophilized to obtain purified polysaccharides.

1. **Physicochemical characterization of Polysaccharides**

The homogeneity and molecular weight were determined by high-performance gel permeation chromatography (HPGPC, Shimadzu Corporation, Kyoto, Japan), with BRT105-104-102 column (8 mm × 300 mm, 35°C) in series and a differential refractive index detector (RID-10A). Ion chromatography (IC) with Dionex ICS3000 equipped with a conductivity detector and a DionexCarboPacTMPA20 analytical column (3 mm × 150 mm) was used to detect the monosaccharide composition.

FT-IR spectra were recorded with a UV-1800 spectrophotometer (Shimadzu Corporation, Tokyo, Japan). Average particle size, polydispersity index (PDI), and ξ-potential of Polysaccharides were determined by the dynamic light scattering instrument (NanoBrook, 90Plus, PALS, U.S). The morphological features of polysaccharides were recorded using a scanning electron microscope (SEM, S-4800, Tokyo, Japan) and atomic force microscope (AFM, Asylum Research, Cypher, USA). Polysaccharides were also analyzed by Shimadzu QP 2010 gas chromatography-mass spectrometry (GC–MS) which fitted with an RXI-5 SIL MS column (30 m × 0.25 mm × 0.25 mm, 120–250°C at 3°C/min, maintained for 5 min) and iron trap MS detector to analyze the glycosidic linkage.

The 1D (1H and DEPT135) and 2D (HSQC, COSY, HMBC, and NOESY) nuclear magnetic resonance (NMR) spectra were collected on a Bruker Advance 500 MHz spectrometer (Brucker, Rheinstetten, Germany) at 500 MHz. X-ray photoelectron spectros-copy (XPS) was performed with a Japan Kratos Axis Ultra HAS spectrometer, the binding energy scale was calibrated with C1s = 284.6 eV. The XRD pattern of EPS was assayed with a D8 Advance X-ray diffractometer (Bruker, Karlsruhe, Germany) The data were collected in the 2θ range 10–80° with tube pressure 40 kV, tube flow 40 mA, and scanning rate 1°/min. The rheological properties of the EPS solution were analyzed by a steady shear test and dynamic sweep measurements using a rotational rheometer with parallel-plate geometry (DHR TA Instruments, USA) at 25℃. Small-angle X-ray scattering (SAXS) data were performed on a Multi-function X-ray polycrystal diffractometer (Nanostar, Bruck, Germany).

1. **Molecular dynamics (MD) simulation**

The GROMACS (version 2018.4) simulation package was used to perform atomistic molecular dynamics simulations. The NAPS*^L.p^* molecules were described using the General Amber force field (Amber99SB), whereas the water molecules were described using the TIP3P water model. The solution system was built by randomly putting 30 polysaccharide chains inside a cubic box of 20 nm×20 nm× 20 nm. After solvation with water molecules, all hydrogen bonds involved were constrained using the LINCS algorithm with an integration step of 2 fs. Electrostatic interactions were calculated using the (Particle-mesh Ewald) PME method. The V-rescale temperature coupling method control was used to set the simulation temperature to 298.15 K and the Parrinello-Rahman method was used to control the pressure at 1 bar, then, the 100 ns at 298.15 K was used for the NVT and NPT equilibrium simulations were then performed at 298.15 K. The visualization of the simulation results was completed using the Gromacs embedded program and VMD.

1. **Preparation of** **Polysaccharides nanoparticles and** **NAPS*^L.p^*@OVA**

For analyzing the self-assembly behaviors of the purified polysaccharides, polysaccharides were induced to self-assemble into nanoparticles. Briefly, the purified polysaccharides were dissolved in deionized (DI) water at 0.8 mg/mL. Then the solution was stirred at room temperature for one hour following lyophilizing to collection.

The NAPS*^L.p^*@OVA, NAPS*^L.p^*@OVA-FITC, and NAPS*^L.p^*@Cy7-OVA were prepared by homogenization methods. In brief, take NAPS*^L.p^*@OVA as an example, 8 mg of NAPS*^L.p^* was dissolved in 10 mL of deionized (DI) water at 200 rpm stirring for 2 h. Subsequently, 10 mg of OVA was dissolved in 10 mL of DI water, followed by the addition of 10 mL of OVA solution with stirring at room temperature. One hour after that, the resulting NAPS*^L.p^*@OVA solution was collected. Unreacted impurities were removed using a 50-kDa ultrafiltration tube, and the supernatant was collected by lyophilizing, NAPS*^L.p^*@OVA was then resuspended with PBS and stored at 4℃. The tube was centrifuged to collect all liquid at the bottom of the tubes for measuring the free OVA using the Enhanced BCA Protein Assay Kit (Solarbio, Beijing, China).

1. **Characterization of NAPS*^L.p^*@OVA**

The size and Zeta potential of NAPS*^L.p^*@OVA were measured with dynamic light scattering (DLS) and Zetasizer Nano ZS (NanoBrook, 90Plus, PALS, U.S.), respectively. A transmission electron microscope (JEOL, JEM-2100, Japan) was performed to observe the morphology of the nanoparticles. The spectros-copy pattern of Fourier transform infrared (FT-IR) was obtained using a spectrometer (Bruker, Karlsruhe, Germany). The ultraviolet−visible (UV−vis) absorption spectra were obtained using a UV−vis spectrophotometer (Shimadzu, Tokyo, Japan), All ﬂuorescence spectra of the samples were measured with a ﬂuorescence spectrometer (Shimadzu, Japan). Circular dichroism spectra were obtained from 190 to 400 nm wavelengths (path length cell: 0.1 cm) on a CD spectrometer (Chirascan, Applied Photophysics, Leatherhead, Surrey, UK). The surface wettability of nanoparticles was analyzed by a three-phase contact angle meter (SL150, USA). To confirm the antigen loading capacity of nanoparticles, NAPS*^L.p^*@OVA were centrifuged at 3000g for 30 min with Thermo ultracentrifuge (Thermo ST8 ST8R, USA), and the supernatant was collected to measure the content of OVA using Enhanced BCA Protein Assay Kit (Solarbio, Beijing, China). The loading capacity of nanoparticles was calculated according to the formula:

Loading capacity= [(Total OVA-Free OVA)/nanoparticles weight] x 100%

The encapsulation efficiency was calculated using the following formula:

Encapsulation efficiency= [(Total OVA - Free OVA) /Total OVA] x 100%

The OVA release behavior from NAPS*^L.p^*@OVA at 37 ℃ was evaluated through the detection of antigens in dialysate after nanovaccines dialysis with a 50-kDa dialysis bag at preset time points.

The crystal structure of the OVA protein was from the RCSB database (http:// www.rcsb. org/). The PDB ID of the OVA protein is 1UHG. The docking simulation was carried out using the auto dock vina program. The geometry of OVA and polysaccharides was optimized using Avogadro software. The docking was performed using the Lamarkian genetic algorithm (LGA). The number of GA runs was set to 100 and the highest populated cluster with the lowest energy conformation based on the scoring function was selected as the binding mode. Among all possible spatial conformations and interaction patterns, the conformation with the lowest energy was selected for visual analysis using Pymol.

1. **Immunity activation and antigen presentation**

In the hemolysis experiment, The OVA, NAPS*^L.p^*, and NAPS*^L.p^*@OVA were mixed with 10% red blood cells (v/v) of 500 µL and incubated at 37°C for 30 min. Pure water and red blood cells were mixed as a 100% hemolysis reference. The supernatant of the sample was centrifuged at 3000 g for 10 min. The absorbance at 540 nm was determined by a UV–Vis spectrometer to calculate the hemolysis rate.

Raw264.7 and BMDCs were seeded in 96-well plates for 12 hours. The OVA, NAPS*^L.p^*, and NAPS*^L.p^*@OVA at various concentrations were added into the culture system and co-cultured for 24 hours, respectively. The cell viability was evaluated by Cell Counting Kit-8 (CCK-8, Biosharp BS350B, China) assay.

BMDC cells are also used for cell uptake experiments. The cells were co-incubated with pre-made FITC-labeled OVA, NAPS*^L.p^*, or NAPS*^L.p^*@OVA at the final concentration of 20 μg/mL for 6 hours. After co-incubation, the Lyso-Tracker Red and DAPI were used to stain lysosome and nuclear, respectively. BMDC were then washed 3 times with PBS in a 35 mm confocal culture dish. Confocal laser scanning microscopy (CLSM, Zeiss LSM 800, Germany) was used to image the cells, and analyzed by Fiji software.

The obtained BMDCs were seeded at a concentration of 1 × 10^6^ cells mL^−1^ in 6-well plates (1 mL per well) and incubated with different treatments for 24 h (OVA concentration was 20 μg/mL). Then BMDCs in each group were collected and divided into flow cytometry tubes, following incubating with antibodies to detect cell surface molecules CD80 (anti-CD80-FITC, anti-CD11c-PE), CD40 (anti-CD40-FITC, anti-CD11c-FITC), MHC II (anti-MHC II-FITC, anti-CD11c-PE), CD86 (anti-CD86-FITC, anti-CD11c-PE), and SIINFEKL-H-2K^b^ (anti- SIINFEKL-H-2K^b^-APC, anti-CD11c-FITC) via flow cytometry, respectively. Furthermore, the cytokines Interleukin (IL-4), cytokines Interleukin (IL-6), cytokines Interleukin (IL-12p70), and tumor necrosis factor (TNF-α) in the supernatant were detected by enzyme-linked immunosorbent assay (ELISA) kits (R&D) to analyze whether BMDCs could be activated by [NAPS*^L.p^*@OVA](mailto:NAPSL.p@OVA).

BMDCs (1 × 10^6^ cells/well) were seeded into culture dishes and cultured at 37 °C overnight, then treated with PBS, LPS (1 μg/mL), and NAPS*^L.p^* (50, 200 μg/mL) for 24 h. Then, the total RNAs were extracted by RNAprep Pure Cell/Bacteria Kit (TaKaRa Bio, Inc.) from cells according to the manufacturer’s instructions. Total RNA solution (1.0 μg/mL) was reverse-transcribed into cDNA using the first strand cDNA synthesis kit (TaKaRa, Beijing, China). RT-PCR was performed on a BIO-RAD CFX96 Connect real-time fluorescence quantification system (BioRad, CA, USA). The relative expression levels of the genes were calculated by the 2^-ΔΔCt^ method, with β-actin serving as the control.

DCs were pretreated with 5 μM TAK-242 (TLR-4 inhibitor, Medchemexpress) for 1 h, followed by the addition of LPS and NAPS*^L.p^* (200 μg/mL) according to the tested concentrations. After 12 h, the supernatants were collected for cytokines assay with TNF-α and IL-6 ELISA kits (R&D) according to the manufacturer's instructions.

The crystal structure of the TLR4 protein was from the RCSB database (http:// www.rcsb. org/). The PDB ID of the TLR4 protein was 2Z62. The docking simulation was carried out using the auto dock vina program. The docking was performed using the Lamarkian genetic algorithm (LGA).

1. **In vivo imaging**

To figure out the pharmacokinetics, fluorescent in vivo imaging was carried out. The OVA was first labeled with fluorescent dye Cy7. Cy7-OVA was used to prepare NAPS*^L.p^*@Cy7-OVA nanoparticles. Then BALB/c mice were treated with Cy7-OVA and NAPS*^L.p^*@Cy7-OVA by i.v. injection, respectively. Mice were anesthetized by isoflurane and imaged by a fluorescence vivo imaging system（Berthold, LB983, Germany）at different time points after injection. The images were then analyzed by Fiji software to compare the fluorescence intensity of the two groups.

1. **Immunization and Antibody Production Assays**

The C57BL/6 mice were assigned randomly to four groups (n=6), PBS group, OVA group, Alum+OVA group, and NAPS*^L.p^*@OVA group. Mice of each group were immunized with samples (containing 50 μg OVA with or without 40 μg NAPS*^L.p^*) on days 0 and 14 through subcutaneous injection. At days 14, 21, and 28 post-primary immunization, the blood was collected via the eye socket. At day 35 post primary immunization, mice were sacrificed and collected major organs for histopathological analysis through H&E staining.

Serum was collected from immunized mice after administration. The total antibody concentration of IgG, IgG1, and IgG2a in serum was measured by ELISA. Brieﬂy, 5 mg/mL OVA was coated onto 96-well ELISA microplates (Corning) at 4°C overnight. After washing with PBST (PBS containing 0.1% Tween 20), 5% BSA-PBS solution was used to block 2 h at 37°C. Then the diluted serum of mice was added into the well and incubated for 2 h at 37°C. Finally, HRP-labeled anti-IgG, anti-IgG1, or anti-IgG2a antibodies were incubated with samples for 2 h. Wash the wells five times with PBST buffer and add TMB solution. Incubate the cells for 15 min then add 50 μL of 0.2 M sulfuric acid to terminate the reaction following measurement titers by the absorbance at 450 nm.

1. **Tumor Prevention against B16-OVA Melanoma Model**

Female C57BL/6 mice at 6-8 weeks were randomly divided into five groups and subcutaneously vaccinated three times at a day -21, -14, and -7. Then on day 0, they were inoculated with 1×10^6^ B16F10-OVA melanoma cells at the right flank of the mouse. The tumor volume (V) was determined by measuring the length (a) and width (b) with calipers every two days and calculated using the formula: V (mm^3^) =1/2*ab^2^. After day 40, mice in each group were fed to calculate survival rates. Mice were sacrificed when the tumor size grew to 1500 mm^3^.

1. **Mice B16-OVA Melanoma model and immunotherapy**

Mice were purchased from Beijing SiPeiFu Biotechnology Co., Ltd. (Beijing, China). The animal protocols complied with the ethical and experiment regulations for animal care at Inner Mongolia University. The animals were maintained under SPF conditions. 1×10^6^ B16F10-OVA cells were subcutaneously inoculated in the right flank of the mice. Mice were randomly divided into five groups (n = 8): PBS, NAPS*^L.p^* (40 μg per mouse), OVA_257-264_ (B16-OVA, 50 μg per mouse), CpG@OVA_257-264_ (10 μg CpG per mouse) and NAPS*^L.p^*@OVA_257-264._ Mice were subcutaneously injected with related drugs on days 4, 11, and 18 after B16F10-OVA tumor administration. The tumor volume (V) was determined by measuring the length (a) and width (b) with calipers every two days and calculated using the formula: V (mm^3^) =1/2*ab^2^. After day 40, mice in each group were fed to calculate survival rates.

Three days after the third vaccination, mice were sacrificed and the tumor tissues of mice were extracted and fixed in 4% paraformaldehyde for histopathological and immunohistochemical analysis. Three adjacent slices of tumor tissues were chosen for H&E staining, CD4, and CD8 immunofluorescence assays.

1. **Flow cytometry analysis of activated immune cells in the B16-OVA tumors**

The tumors were collected after euthanasia of the mice and digested into single-cell suspensions for flow cytometric analysis. Briefly, the obtained single-cell suspensions were washed by PBS three times, and collection the pellet by centrifugation. The single cells were incubated with anti-CD3-APC, anti-CD4-FITC, and anti-CD8a-FITC (CD4 and CD8 T cells), anti-Foxp3-APC, anti-CD25-PE, anti-CD11b-FITC, and anti-Ly6G(Gr1)-PE. The flow cytometric analysis was conducted using a NovoCyte Flow Cytometer (ACEA Biosciences, Inc). 100000 events were collected and analyzed through CytoExpert 2.0 software.

Splenocytes were obtained and digested to single-cell suspensions for flow cytometry 24 hours after the last vaccination. After washing with PBS, the single cells were stained with anti-CD3-APC, anti-CD4-FITC, and anti-CD8a-FITC (CD4 and CD8 T cells). The single cells were treated with red cell lysis buffer for 5 min to remove red cells. After washing with RPMI 1640 medium, splenocytes were seeded in 24-well plates, where splenocytes were restimulated with OVA_257-264_ (10 μg/mL) for 3 days. Splenocytes were collected and washed with PBS. After that, cells were stained with anti-mouse-CD3-APC, anti-mouse-CD4-FITC, and anti-mouse-CD8-FITC on ice for 30 min. Then, the cells were fixed, permeabilized, and stained with anti-mouse-IFN-γ-PE for intracellular IFN-γ. Flow cytometric analysis was conducted using a NovoCyte Flow Cytometer and analyzed using the software Novo express (ACEA Biosciences, Inc).

For immune memory investigation, C57BL/6 mice were immunized as described above. On day 60, splenocytes were harvested from the vaccinated mice and stained with anti-CD3-APC, anti-CD8-FITC, anti-CD44-PE, anti-CD62L-PE/Cy7, and anti-CD69-PE antibodies according to the manufacturer’s protocols for flow cytometry.

1. **NAPS*^L.p^*-based vaccination offers a strong influenza protection**

Preparation of H1N1 WIV and NAPS*^L.p^*@H1N1 WIV complexes: H1N1 influenza virus (A/Puerto Rico/8/34) were identified and stored by our laboratory, and then the viruses were purified using discontinuous sucrose density gradient centrifugation. Inactivated viruses were tested for complete loss of infectivity by inoculation into 10-day-old specific-pathogen-free (SPF) embryonated eggs for three passages. The quantity of viruses was measured by a BCA protein assay kit. The HA protein concentration was about 35% of the total protein. H1N1 WIV and NAPS*^L.p^* were dissolved in phosphate-buffered saline (PBS, 0.01 M) to adjust the concentration to 100 μg/mL and 1 mg/mL, respectively. H1N1 WIV and NAPS*^L.p^* complexes were prepared by adding H1N1 WIV solution into NAPS*^L.p^* solution at equal volume and vortexed for 0.5 h at room temperature.

Immunogenicity Study: 6-week-old C57BL/6 mice were divided into five groups (n = 12 per group) and immunized intranasally at 0 and 14 days with 200 µL PBS or NAPS*^L.p^* (50 µg) alone, or H1N1 WIV (containing 5 µg HA) alone, or H1N1 WIV (containing 5 µg HA) plus AlOH_3_ (50 µg), or H1N1 WIV (containing 5 µg HA) and NAPS*^L.p^* (50 µg) complexes, respectively. The serum was collected on days 14, 21, and 28 after the first immunization. Sera were tested for antibodies against H1N1 viruses using the Hemagglutination Inhibition (HI) test. H1N1 WIV-specific serum IgG antibodies were determined by indirect ELISA.

Virus Challenge: 28 days post-primary immunization, vaccinated mice were anesthetized and then inoculated intranasally challenged with 10^6^ CFU of the H1N1 influenza virus. Body weight changes and survival rates of infected mice (n = 9 per group) were monitored for 15 days. Lung tissues (n = 3 per group) were collected for pathological or histopathological examination, or virus titration (n = 3 per group) at 5 days post-infection (p.i.).

For detection of viral titers, each tissue sample was homogenized in 1 mL of PBS and centrifuged at 10000 rpm for 10 min, and 0.1 mL of supernatant was used to inoculate confluent Madin Darby canine kidney (MDCK) cell monolayers by using 96-well plates under from initial dilutions of 1:10. Afterward, the supernatants were replaced with medium and incubated at 37 °C for 72 h. The presence of the virus in the supernatant was assayed by measuring the hemagglutinating activity.

1. **Statistical analysis**

All data were presented as means ± standard deviations. Statistical significance was analyzed by one-way analysis of variance (ANOVA) using the GraphPad Prism 8 (San Diego, USA). For analyses of data with two independent variables, the statistical significance of all tests was defined as *, p< 0.05，**, p<0.01,***, p<0.001.

**Supplementary Table 1.** Screened bacteria and FTIR of isolated EPSs.


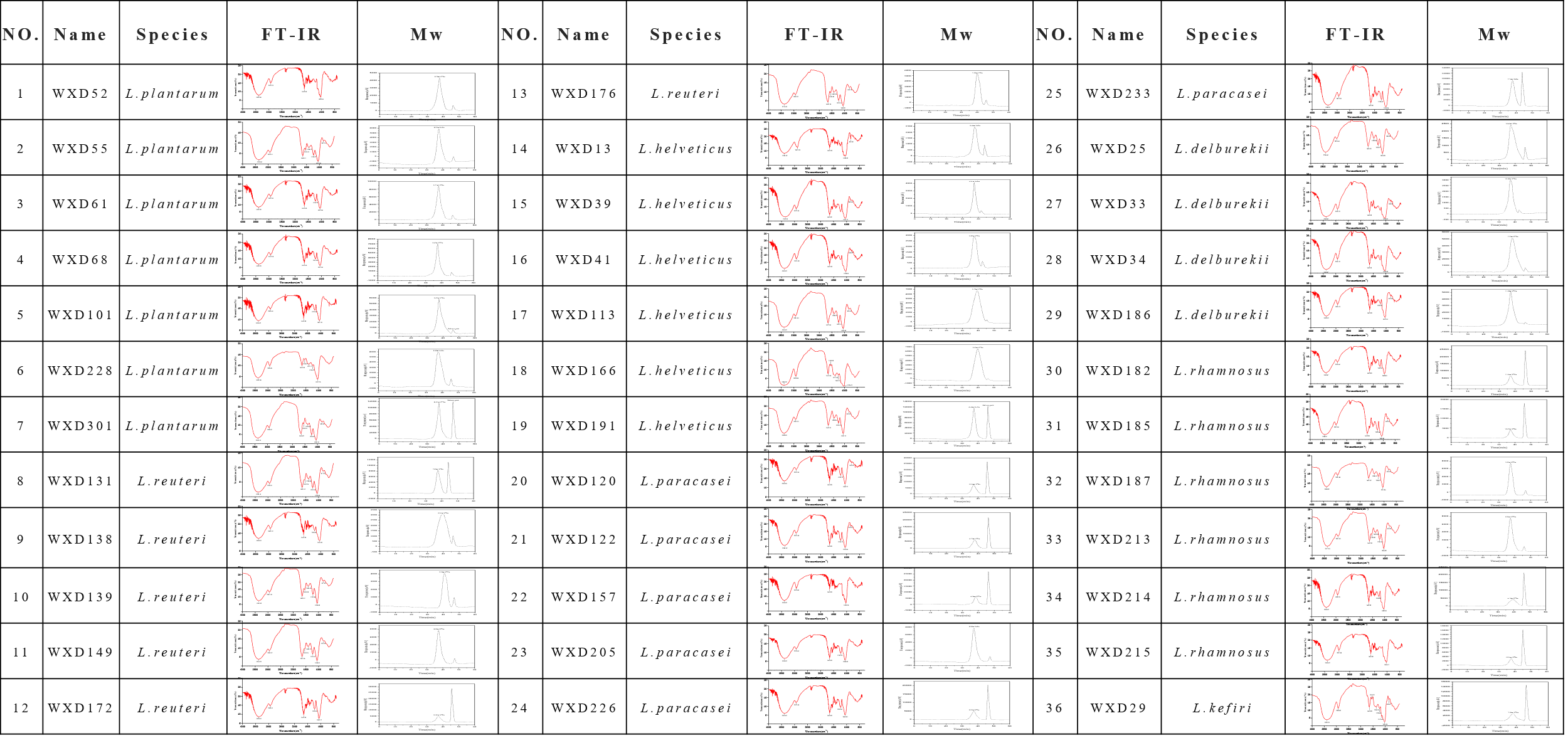


**Supplementary Table 2.** The physical and chemical properties of exopolysaccharides


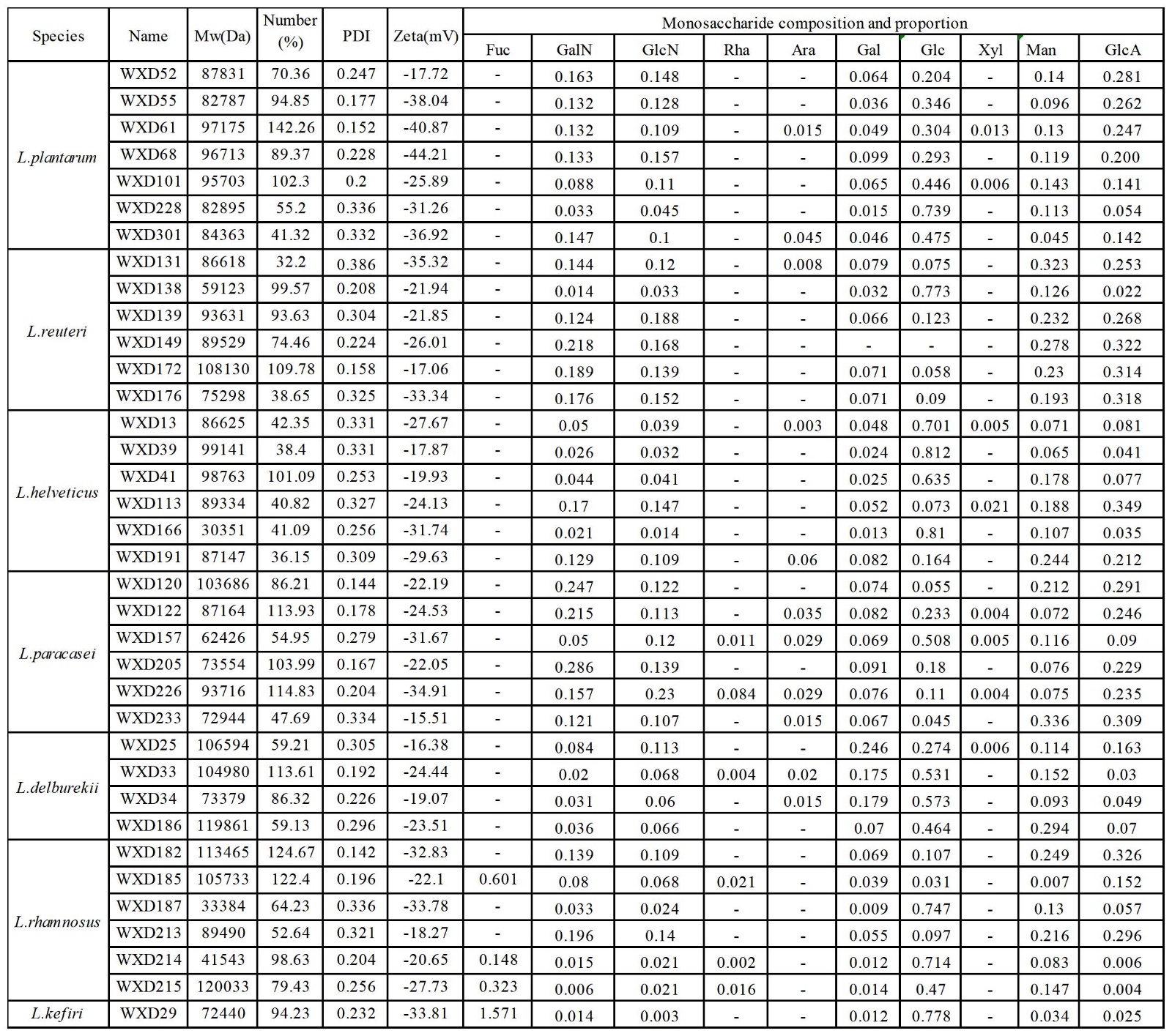


**Supplementary Table 3.** The results of partially methylated alditol acetate (PMAA) of NAPS*^L.p^*

| **RT** | **Methylated sugar** | **Mass fragments(m/z)** | **Molar ratio** | **Type of linkage** |
| --- | --- | --- | --- | --- |
| 16.894 | 2,3,5-Me3-Araf | 43,71,87,101,117,129,145,161 | 0.064 | Araf-(1→ |
| 22.44 | 2,3-Me2-Araf | 43,71,87,99,101,117,129,161,189 | 0.056 | →5)-Araf-(1→ |
| 22.759 | 2,3-Me2-Arap | 43,87,101,117,129,161,189,217 | 0.012 | →4)-Arap-(1→ |
| 24.762 | 2,3,4,6-Me4-Manp | 43,71,87,101,117,129,145,161,205 | 0.166 | Manp-(1→ |
| 25.856 | 2,3,4,6-Me4-Manp | 43,71,87,101,117,129,145,161,205 | 0.010 | Galp-(1→ |
| 29.722 | 3,4,6-Me3-Manp | 43,87,129,161,189 | 0.192 | →2)-Manp-(1→ |
| 30.206 | 2,3,6-Me3-Galp | 43,87,99,101,113,117,129,131,161,173,233 | 0.032 | →4)-Galp-(1→ |
| 30.655 | 2,3,6-Me3-Glcp | 43,87,99,101,113,117,129,131,161,173,233 | 0.155 | →4)-Glcp-(1→ |
| 31.191 | 2,4,6-Me3-Manp | 43,71,85,87,99,101,117,129,161 | 0.017 | →3)-Manp-(1→ |
| 31.9 | 2,3,4-Me3-Manp | 43,71,87,99,101,117,129,159,161 | 0.247 | →6)-Manp-(1→ |
| 38.092 | 3,4-Me2-Manp | 43,87,99,129,189 | 0.034 | →2,6)-Manp-(1→ |
| 40.009 | 2,4-Me2-Galp | 43,87,117,129,159,189,233 | 0.015 | →3,6)-Galp-(1→ |

**Supplementary Table 4.** ^1^H and ^13^C signals assignments of NAPS*^L.p^*

| **Glycosyl residues** | **H1/C1** | **H2/C2** | **H3/C3** | **H4/C4** | **H5/C5** | **H6a/C6** | **H6b/C-Me** | **H6b/C=O** |
| --- | --- | --- | --- | --- | --- | --- | --- | --- |
| →2,6-Man-1→ | 5.00/99.57 | 3.93/80.07 | 3.81/71.53 | 3.55/68.01 | 3.73/74.47 | 3.91/67.15 | 3.66 |  |
| →6-Man-1→ | 4.8/100.78 | 3.89/71.37 | 3.75/71.9 | 3.6/67.98 | 3.73/74.48 | 3.83/67.15 | 3.66 |  |
| →2-Man-1→ | 5.18/101.84 | 4.01/79.62 | 3.81/71.57 | 3.74/67.69 | 3.6/74.58 | 3.64/62.11 | 3.77 |  |
| →3-Man-1→ | 5.04/103.49 | 3.98/71.5 | 3.9/79.25 | 3.55/68.35 | 3.73/74.7 | 3.64/62.11 | 3.77 |  |
| →Man-1→ | 4.95/103.38 | 4.25/71.49 | 3.73/71.77 | 3.61/67.82 | 3.69/74.66 | 3.64/62.11 | 3.77 |  |
| →4)-β-D-GlcpA-(1→ | 4.39/105.45 | 3.27/73.67 | 3.49/75.09 | 3.63/82.37 | 3.59/77.67 | 0.02/175.67 |  |  |
| →3)-β-D-GlcpNac-(1→ | 4.45/102.54 | 3.91/52.27 | 3.75/81.18 | 4.12/68.83 | 3.87/73.88 | 3.68/62.34 | 23.88 | 176.31 |
| →6)-α-D-Glcp-(1→ | 4.91/99.1 | 3.53/72.73 | 3.67/74.63 | 3.47/70.87 | 3.86/71.47 | 4.01/67.64 | 3.91 |  |

**Supplementary Table 5.** The Size and Zeta Potential Measurement of NAPS*^L.p^*, OVA and NAPS*^L.p^*@OVA

| Sample | Size(nm) | PDI | Zeta (mV) | EE (%) | LC (%) |
| --- | --- | --- | --- | --- | --- |
| NAPS*^L.p^* | 41.32±3.14 | 0.121±0.04 | -36.92±0.15 | - | - |
| OVA | 125.67±0.90 | 0.143±0.01 | -20.82±0.30 | - | - |
| NAPS*^L.p^*@OVA | 83.98±1.62 | 0.142±0.06 | -17.01±0.90 | 87.30±2.64 | 57.98±1.65 |


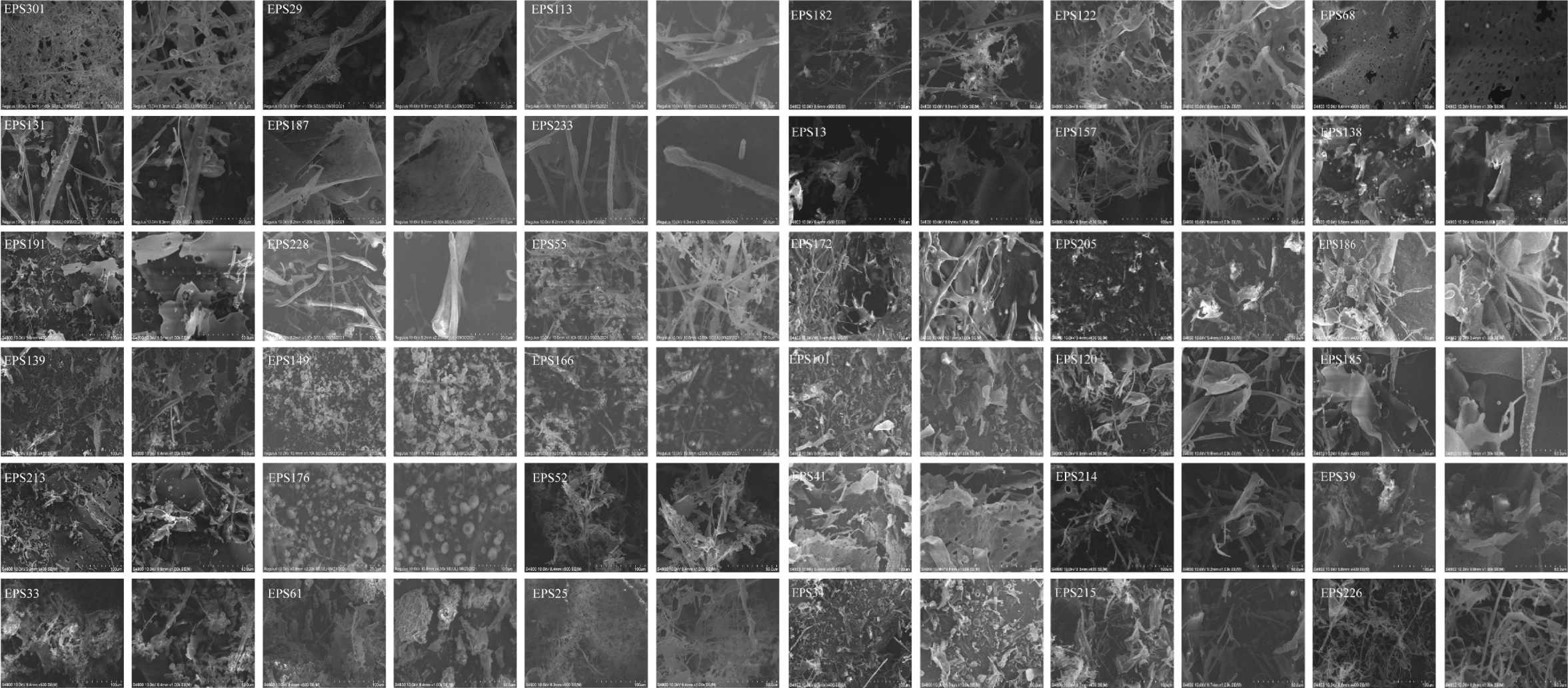


**Supplementary figure 1.** SEM images of isolated EPSs from screened bacteria.

**Supplementary figure 2.** UV spectra of NAPS*^L.p^* in the range of 180–600 nm


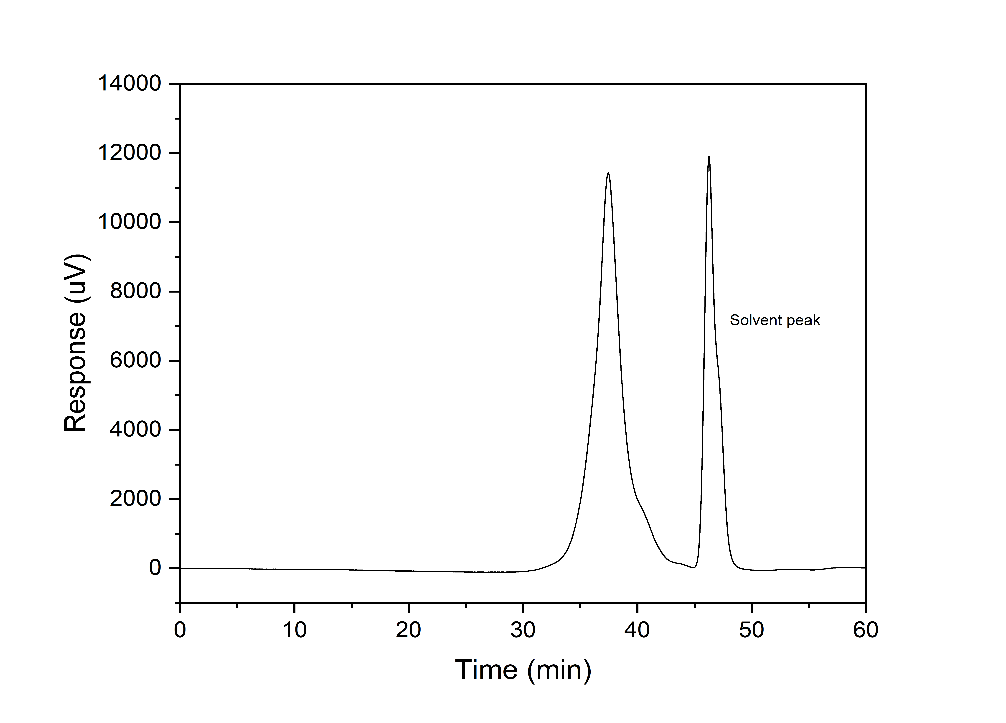


**Supplementary figure 3.** The molecular weight distribution of NAPS*^L.p^*


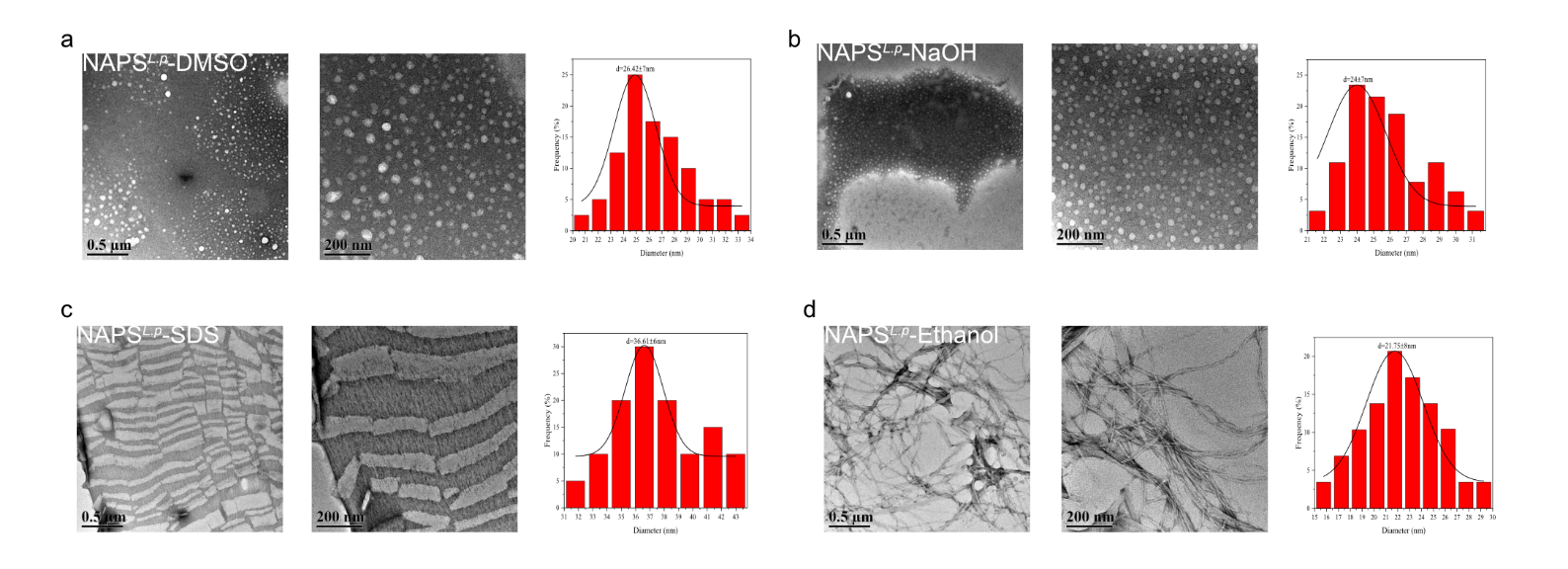


**Supplementary figure 4.** TEM images of NAPS*^L.p^*. (a) The images of NAPS*^L.p^*-SDS; (b) the images of NAPS*^L.p^* -DMSO;(c) the images of NAPS*^L.p^*-NaOH; (d) the images of NAPS*^L.p^*-Ethanol.


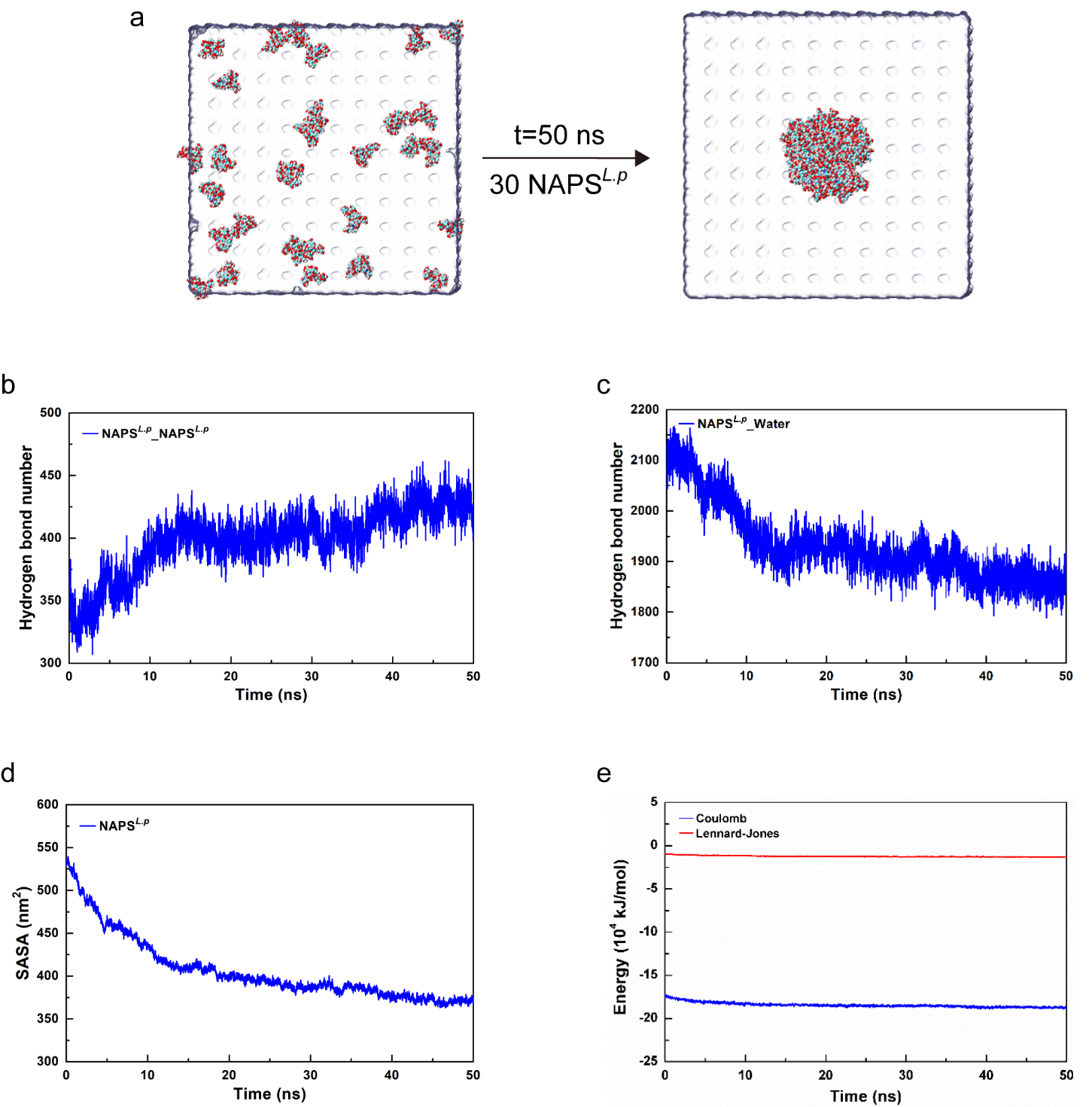


**Supplementary Figure 5.** (a). Snapshots of the MD simulation systems at 50 ns. (b). The number of intermolecular hydrogen bonds between NAPS*^L.p^* molecules. (c). The number of intermolecular hydrogen bonds between NAPS*^L.p^* molecules and water molecules. (d). The change of SASA. (e). The energy of Coulomb and LJ-Potential.

All 30 NAPS*^L.p^* molecules clustered together to form a stable spherical nanocluster at 50 ns (**Supplementary Fig. 5a**). This phenomenon confirmed the spontaneous aggregation behavior of NAPS*^L.p^* molecules in an aqueous solution.

Subsequently, to analyze the role of electrostatic interaction and van der Waals interaction in the self-assembly process of NAPS*^L.p^* molecules, the interaction energy between NAPS*^L.p^* molecules were calculated by the gmx energy module of the Gromacs. The results were shown in **Supplementary Figure 5c and e**, the NAPS*^L.p^* intermolecular Lennard-Jones potential (LJ-Potential) and Coulomb energy contributions were -184284.897 kJ/mol and -12253.911 kJ/mol respectively. Coul and LJ between NAPS*^L.p^* molecules and water molecules are -62899.067 kJ/mol and -4930.415kJ/mol, respectively. During the aggregation process of NAPS*^L.p^*, the LJ-Potential remained stable from 0 to 50 ns, while the Coulomb maintained a stable trend after a significant increase in the first 4 ns. These results revealed the van der Waals forces and electrostatic in­teractions were not the main factors that enable NAPS*^L.p^* to self-assemble.

**Supplementary figure 6.** CD spectroscopy analysis of NAPS*^L.p^*

**Supplementary figure 7.** The monosaccharide components of NAPS*^L.p^*

**Supplementary figure 8.** FT-IR spectra of NAPS*^L.p^* in the range of 600–4000 cm^−1^.

Its absorption peaks are distributed in a range of 4000–400 cm^-1^. A brand nearby 3384.14 cm^-1^ indicated the stretching vibration of hydroxyl groups. The weak absorption peak at around 2939.51 cm^-1^ represented a C-H stretching vibration, while the band at 1656.82 cm^-1^ was ascribed to the stretching vibrations of C=O, respectively. The peak at 1384.38 cm^-1^ was on behalf of the C-H deformation vibration, and the peak at 1230.58 cm^-1^ was assigned to the vibrations of the O-H bonds. In addition, the absorption at 1053.13 cm^-1^ indicated the presence of C-O bonds, while the weak bands near at 825.53 cm^-1^ demonstrated that glucosyl residues could be present in β-configurations.


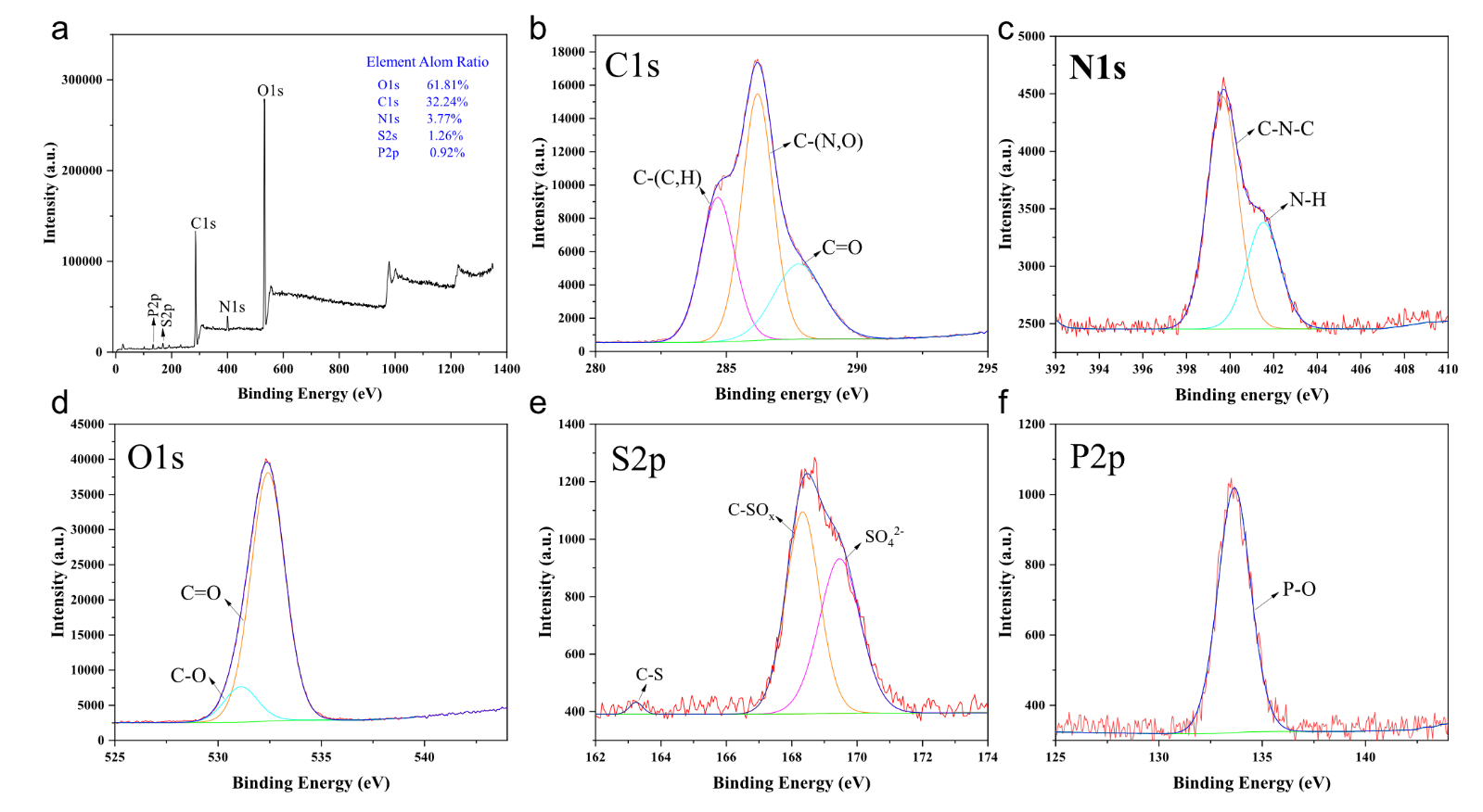


**Supplementary figure 9.** XPS spectroscopy analysis of NAPS*^L.p^* (a). The high-resolution XPS peaks of C1s (b), N1s (c), O1s (d), S2p (e), P2p (f).

NAPS*^L.p^* contains 61.81% O, 32.24% C, 3.77% N, 1.26% S, and 0.92% P as analyzed by XPS **(Supplementary Fig. 9a)**, indicating that there are other functional groups in NAPS*^L.p^* in addition to the mono-saccharides that contain only C, H, and O elements (the H element cannot be detected by XPS). These results conﬁrmed that the 1656.82 cm^−1^ peak in the ATR−FTIR spectra of NAPS*^L.p^* **(Supplementary Fig. 8)** was due to the presence of both the amido or amino groups and the carboxylic group in the NAPS*^L.p^* sample, implying that NAPS*^L.p^* is acetylated and simultaneously carbonylated. In the high-resolution of the C1s spectrum **(Supplementary Fig. 9b)**, peaks at 284.4, 286.8, and 288.4 eV can be assigned to groups C− (C, H), C− (N, O), and C=O, respectively. The N1s peaks **(Supplementary Fig. 9c)** at 399.7 and 401.4 eV could separately be ascribed to amide nitrogen C−N−C and the amino nitrogen N−H. The O1s peak **(Supplementary Fig. 9d)** at 530.3 eV belongs to C−O. The other O1s peak at 532.6 eV is ascribed to the C=O group. As for S2p **(Supplementary Fig. 9e)**, the sulfur is mainly in −C−S− (165.3 eV), sulfonic acid R−SO_X_ (169.4 eV), and SO_4_^2-^ (170.7 eV), demonstrating that NAPS*^L.p^* is a sulfated polysaccharide. Moreover, the P2p peak **(Supplementary Fig. 9f)** located at 134.8 eV can be assigned to the form of P−O, which mainly comes from the phosphate group. Overall, the results demonstrated that NAPS*^L.p^* is acetylated, carboxylated, phosphorylated, and sulfated. Modiﬁcations, like acylation, methylation, sulfation, epimerization, and phosphorylation, can occur at various positions within a NAPS*^L.p^* both naturally or by chemical methods, and these can affect their physicochemical properties and bioactivities.


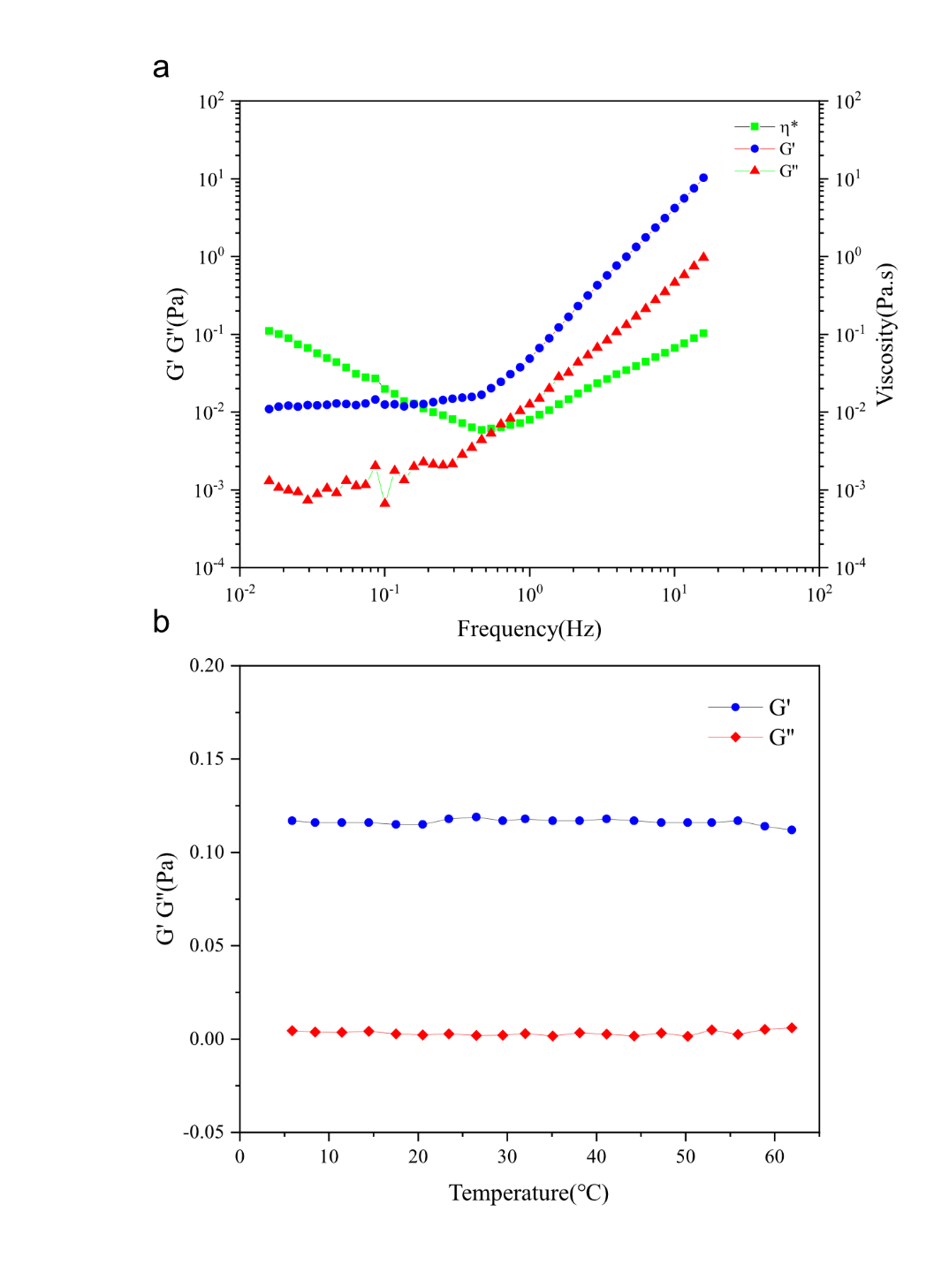


**Supplementary figure 10.** The rheological analysis of NAPS*^L.p^*. (a) Strain sweep at 0.01 Hz and 100 Hz in the liquid at a temperature of 25℃ and a frequency of rad/s shown as G′, G′′ and ɳ*. (b) Strain sweeps at a temperature of 0℃ from 65℃ shown as G′, G′′.

With the aim of knowing the mechanical properties of NAPS*^L.p^*, the rheometer test was performed to investigate the relationship between the viscosity (η) of the NAPS*^L.p^* aqueous solution and the shear rate in the steady shear measurement, the relationship between the storage moduli (G′) and the loss moduli (G″) of the NAPS*^L.p^* aqueous solution, and the stability of G′ and G′′ over the entire test temperature range of 5-65°C. It can be seen from **Supplementary Figure 10a** that linear oscillatory shear rheology showed that NAPS*^L.p^* maintained solid-like (G′ > G″) properties over the tested frequency range. In addition, the viscosity (η) of NAPS*^L.p^* aqueous solution decreases with increasing shear frequency in the range of 0.01 to 1 Hz. However, the NAPS*^L.p^* aqueous solution exhibited shear-thinning behavior instead. This result may be due to the destruction of the rigid molecular structure of raw NAPS*^L.p^*, thereby revealing shear-thinning behavior. Moreover, In the range of 1 to 100 Hz, the viscosity (η) of NAPS*^L.p^* aqueous solution increases with increasing shear frequency, a stage in which NAPS*^L.p^* forms a stable conformation. In addition, it can be seen from Figure S8b that the storage modulus (G′) > loss modulus (G″) of the EPS aqueous solution in the temperature range of 5-65°C (**Supplementary Fig. 10b**), indicating that the NAPS*^L.p^* solution exhibits gel behavior and is thermally stable sex.


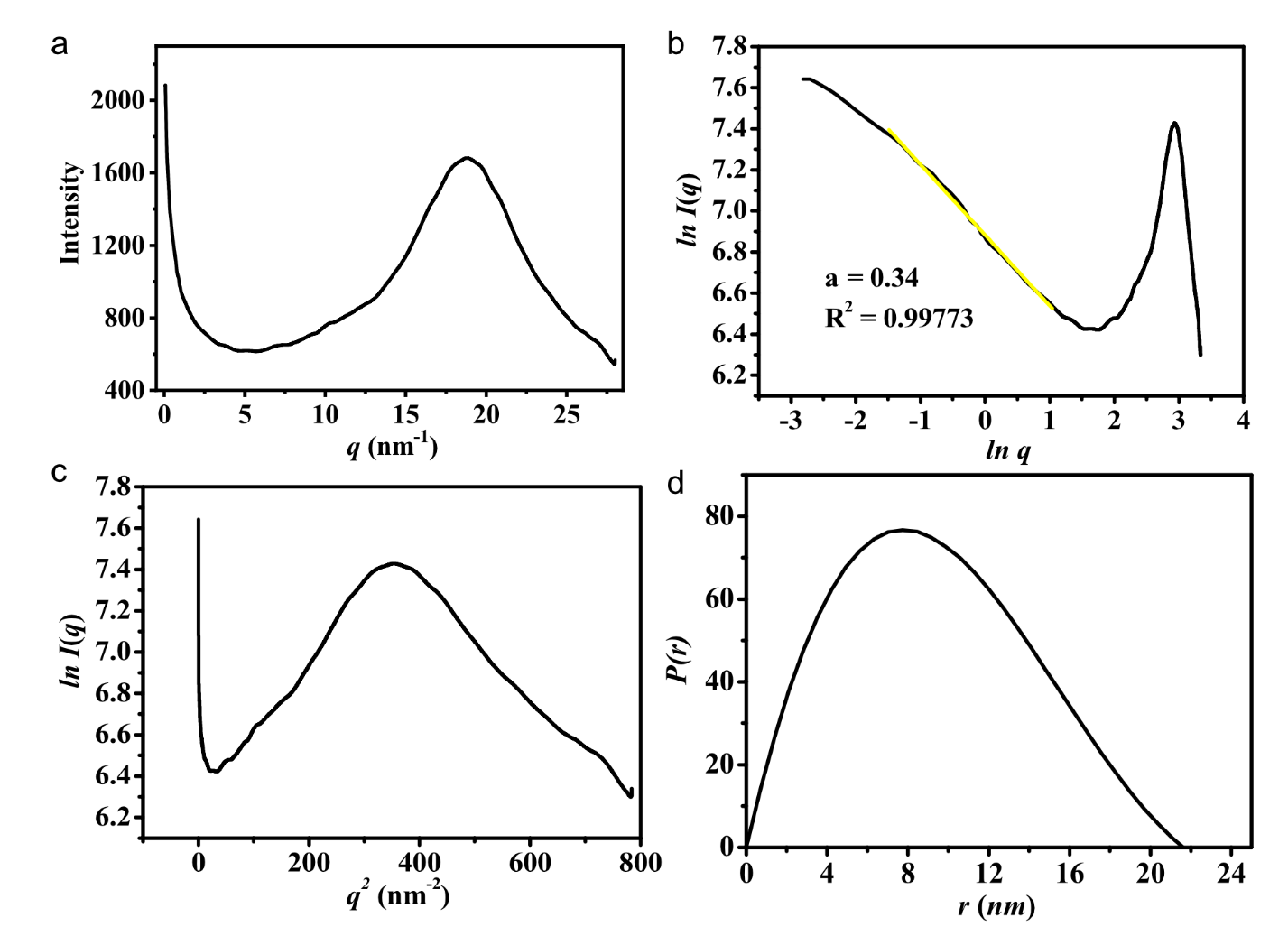


**Supplementary figure 11.** (a). The scattering curve of NAPS*^L.p^*; (b). the fractal features of NAPS*^L.p^*; (c). the Guinier curve of NAPS*^L.p^*; (d). the pair distance distribution function of NAPS*^L.p^*

In order to gain better insight into the nanoscale microstructure, SAXS experiments were performed (**Supplementary Fig. 11**). The SAXS curves showed a typical small angle scattering behavior, which indicated the existence of uniform electron density scatterers at the nanometer scale (**Supplementary** **Fig. 11a**). The fractal characteristic map was obtained by fitting the scattering curve (**Supplementary** **Fig. 11b**). The fractal coefficient of NAPS*^L.p^* was α=0.34, which indicated that the polysaccharide system was relatively regular. From the Guinier curves (**Supplementary** **Fig. 11c**), the relationship of ln I(q)-q^2^ was a concave curve with almost no straight-line segments in the q^2^ region (0 nm < q^2^ < 800 nm), which meant that the sample had a spherical polydisperse morphology. The shape and size information of NAPS*^L.p^* can be obtained by comparing the distance distribution curve (**Supplementary** **Fig. 11d**), the distance distribution function of NAPS*^L.p^* is symmetrical, indicating that the morphology of NAPS*^L.p^* is a spheroid. The intersection value of the curve and the abscissa represents the particle size, which is 20.18 nm.


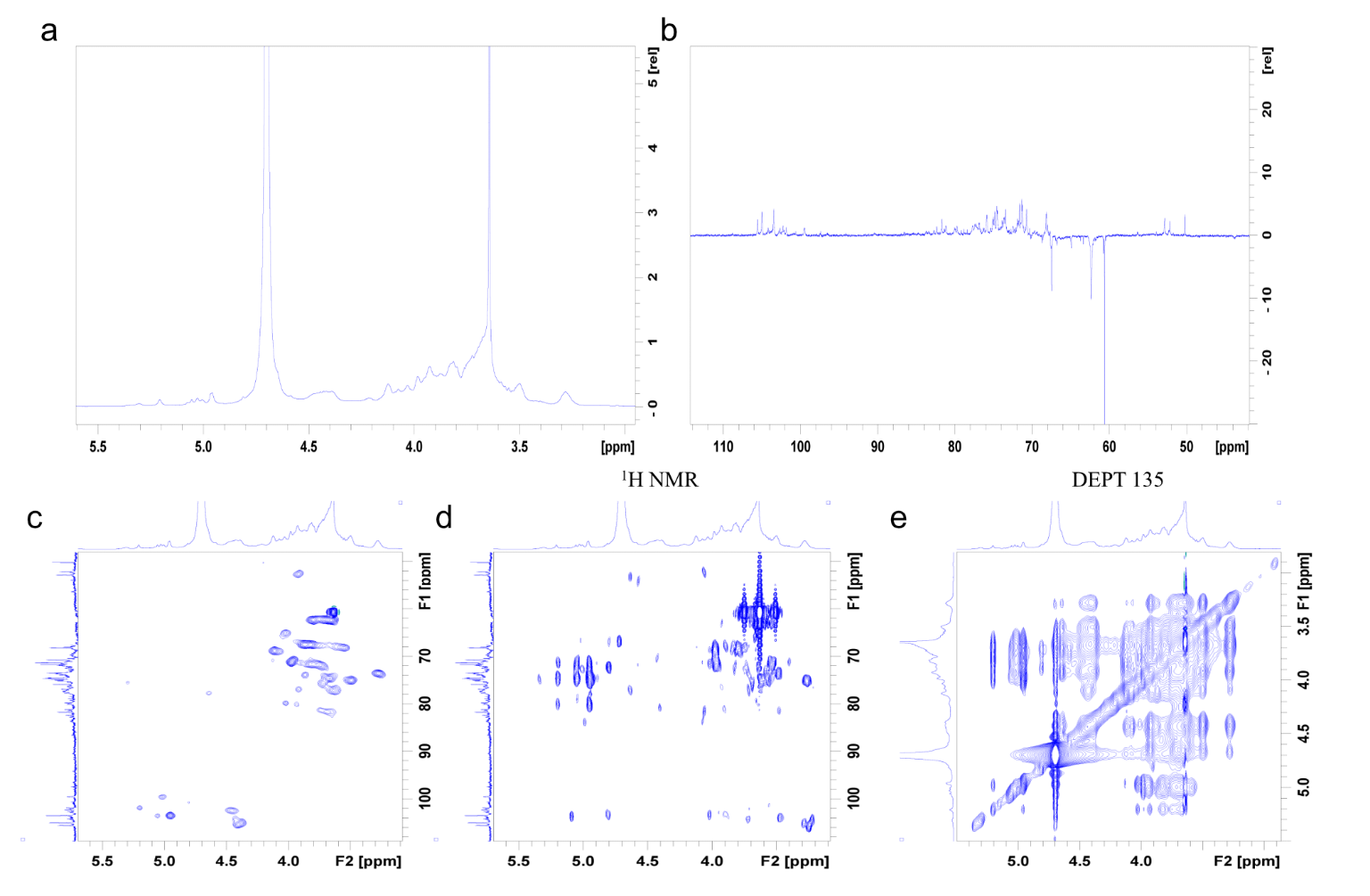


**Supplementary figure 12.** NMR spectra of NAPS*^L.p^*. (a) ^1^H NMR spectrum; (b) DEPT135 spectrum; (c) HSQC spectrum; (d) HMBC spectrum; (e) NOESY spectrum.

The ^1^H NMR spectrum of NAPS*^L.p^* **(Supplementary Fig. 12a)** showed proton spectrum signals were mainly concentrated between 3.0 and 5.5 ppm. δ3.2-4.0 ppm was the sugar ring proton signal, in addition, the main end group proton peaks were concentrated in the δ4.3-5.5 ppm area. The DEPT135 spectrum of NAPS*^L.p^* **(Supplementary Fig. 12b)** showed that the main anomeric carbon signals were mainly between 60 to 120 ppm. The main signal peaks were distributed in the 20-180 ppm region in the DEPT135 carbon spectrum. The main anomeric carbon signal peaks δ99.1, 99.57, 100.78、101.84、102.54、103.38、103.49、and 105.45 are mainly in δ93-105. In the DEPT135 spectrum, C6 signal peaks were mainly distributed in the high field of δ 60-70 ppm, 68.85, 67.06, 67.47, 62.16, 61.89, 60.62 ppm peaks were inverted, indicating the chemical shift of C6. According to the HSQC spectrum (**Supplementary Fig. 12c**), the anomeric carbon signal was δ102.57, and the corresponding anomeric hydrogen signal was δ4.47, respectively. In the 1H-1H-COSY spectrum (**Fig. 2b**), the signals of H1-2, and H2-3 were 4.47/3.93 and 3.93/3.77, respectively. H1, H2, and H3 were identified at δ4.47, 3.93, and 3.77, respectively, and the corresponding C1-3 were 102.57, 52.3, and 81.21. The signals of C6 were δ62.37, and the corresponding H6 was 3.7. The signals of H6-5, and H5-4 were 3.7/3.89 and 3.89/4.41, and the corresponding C4-6 were δ68.85, 73.90, and 62.36, respectively. The chemical shift of the carbonyl carbon of the acetyl group is δ176.33 and the chemical shift of the acetyl group is δ23.90. Therefore, this glycosidic bond is presumed to be →3)-β-D-GlcpNAc-(1→. According to HMBC and NOESY (**Supplementary Fig. 12d and Fig. 12e**), all glycosidic bond signals were speculated (**Supplementary Table 4**). There was a correlation peak between the anomer hydrogen of →3-Man-(1→and C2 of glycosidic bond→2)-Man-(1→, indicating the existence of glycosidic bond →3-Man-1→2-Man-1→. In the same way, the correlation peaks between the anomer hydrogen of →2-Man-(1→and C2 of glycosidic bond→2,6)-Man-(1→, →2,6-Man-(1→and C2 of glycosidic bond→2,6)-Man-(1→, →4)- β-D-GlcpA-(1→and C3 of glycosidic bond→3)-β-D-GlcpNAc-(1→, →3)-β-D-GlcpNAc-(1→and H4 of glycosidic bond→4)-β-D-GlcpA-(1→, →4)-β-D- GlcpA-(1→and H3 of glycosidic bond→3)-Man-1→, Man-1→(6→and H6 of glycosidic bond→6)-α-D-Glcp-(1→, →6-α-D-Glcp-(1→and H6 of glycosidic bond→2,6)-α-Man-(1→, Man-1→and H6b of glycosidic bond→6-Man-1→, and →6-Man-1→and H6b of glycosidic bond→2,6- Man-1→, which indicated the existence of glycosidic bond →2-Man-1→2,6-Man-1→, →2,6-Man-1→2,6-Man-1→, →4)-β-D-GlcpA-(1→3)-β-D-GlcpNAc-(1→, →3)-β-D-GlcpNAc-(1→4)- β-D-GlcpA-(1 →, →4)-β-D-GlcpA- (1→3-Man-1→, Man-1→6)-α-D-Glcp-(1→, Man-1→2,6 -Man-1→, Man-1→6-Man-1→, and →6-Man-1→2,6-Man-1→


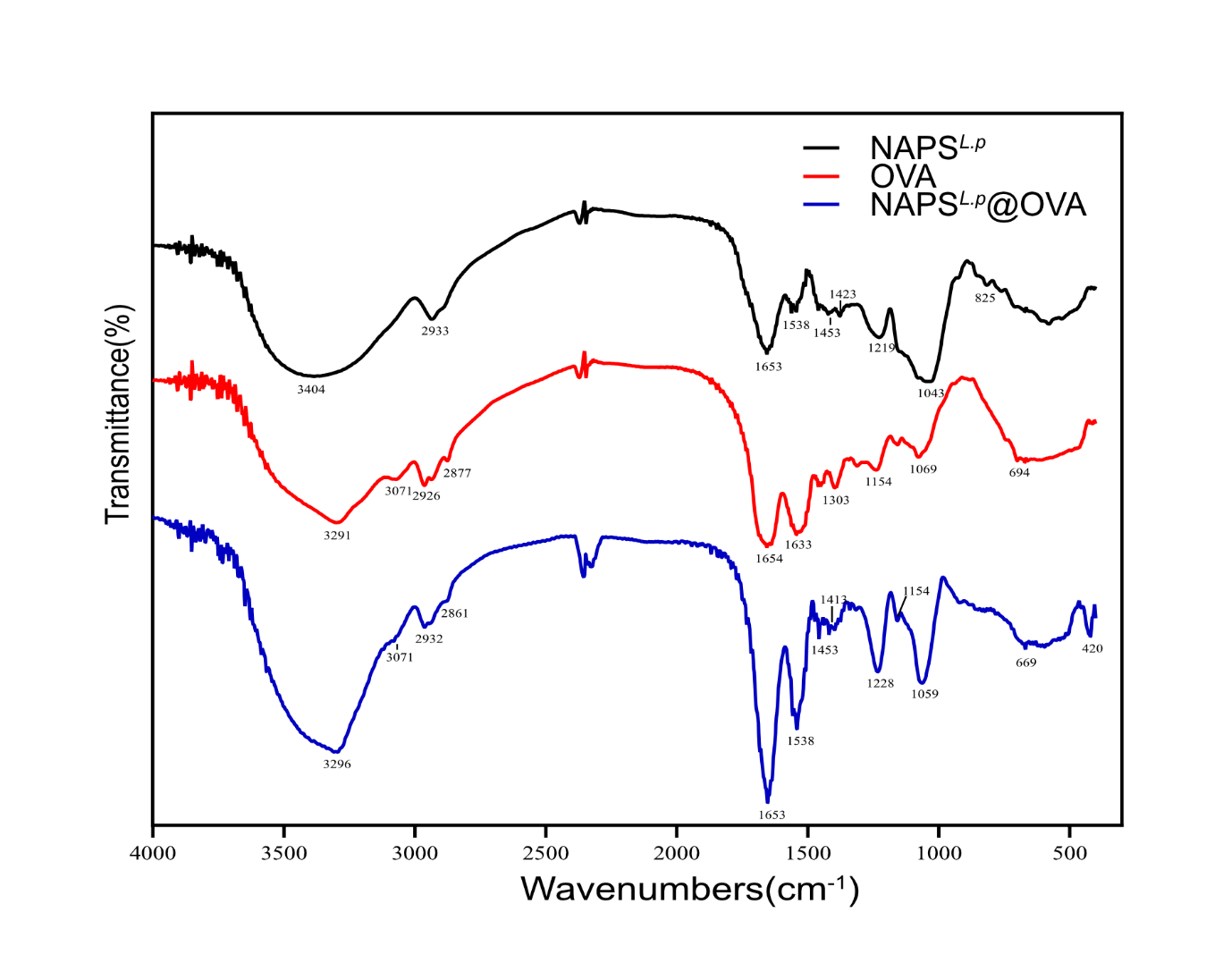


**Supplementary figure 13.** FT-IR spectra of NAPS*^L.p^*, OVA, and NAPS*^L.p^*@OVA in the range of 600–4000 cm^−1^.

**Supplementary figure 14.** CD spectroscopy analysis of OVA, NAPS*^L.p^*, and NAPS*^L.p^*@OVA.


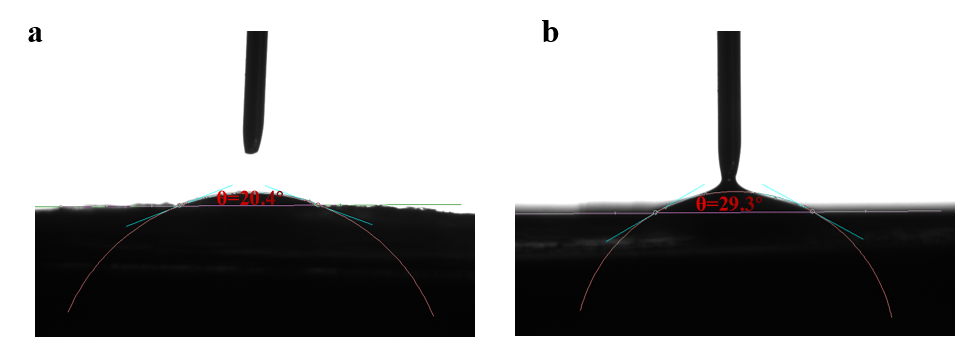


**Supplementary figure 15.** (a). The contact angle analysis of NAPS*^L.p^*; (b). The contact angle analysis of NAPS*^L.p^*@OVA.

**
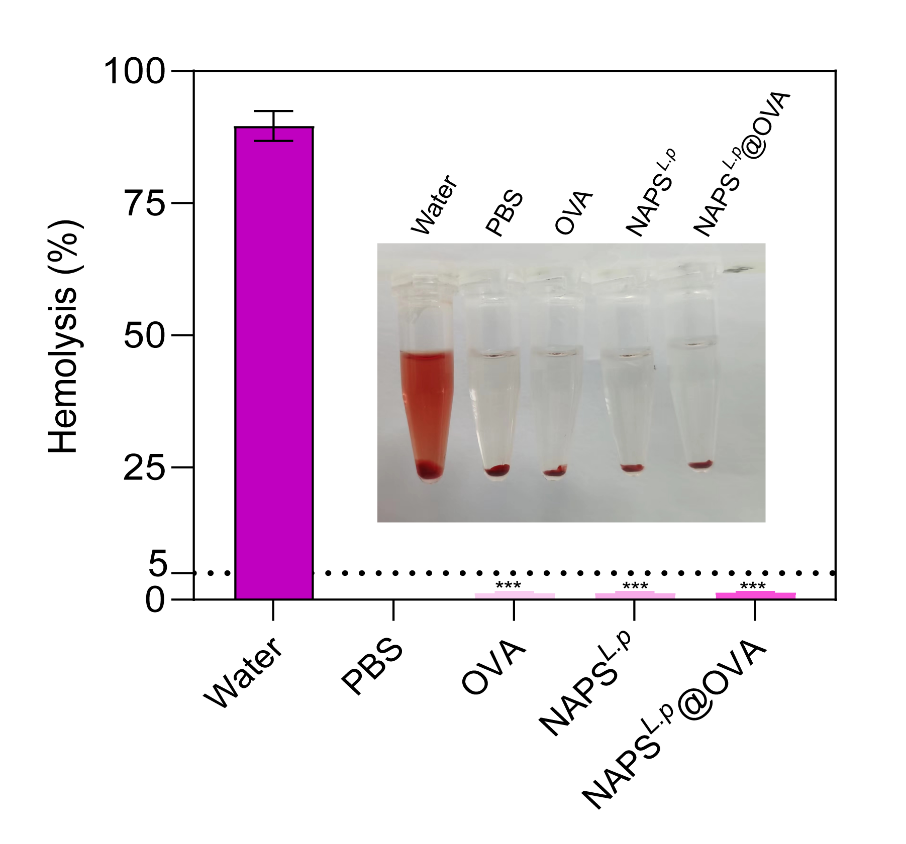
**

**Supplementary figure 16.** In vitro hemolytic performance evaluation of OVA, NAPS*^L.p^*, and NAPS*^L.p^*@OVA.

**Supplementary figure 17.** Cytotoxicity of RAW264.7 after coculturing for 24h with NAPS*^L.p^*, OVA and NAPS*^L.p^*@OVA (n=3).

**
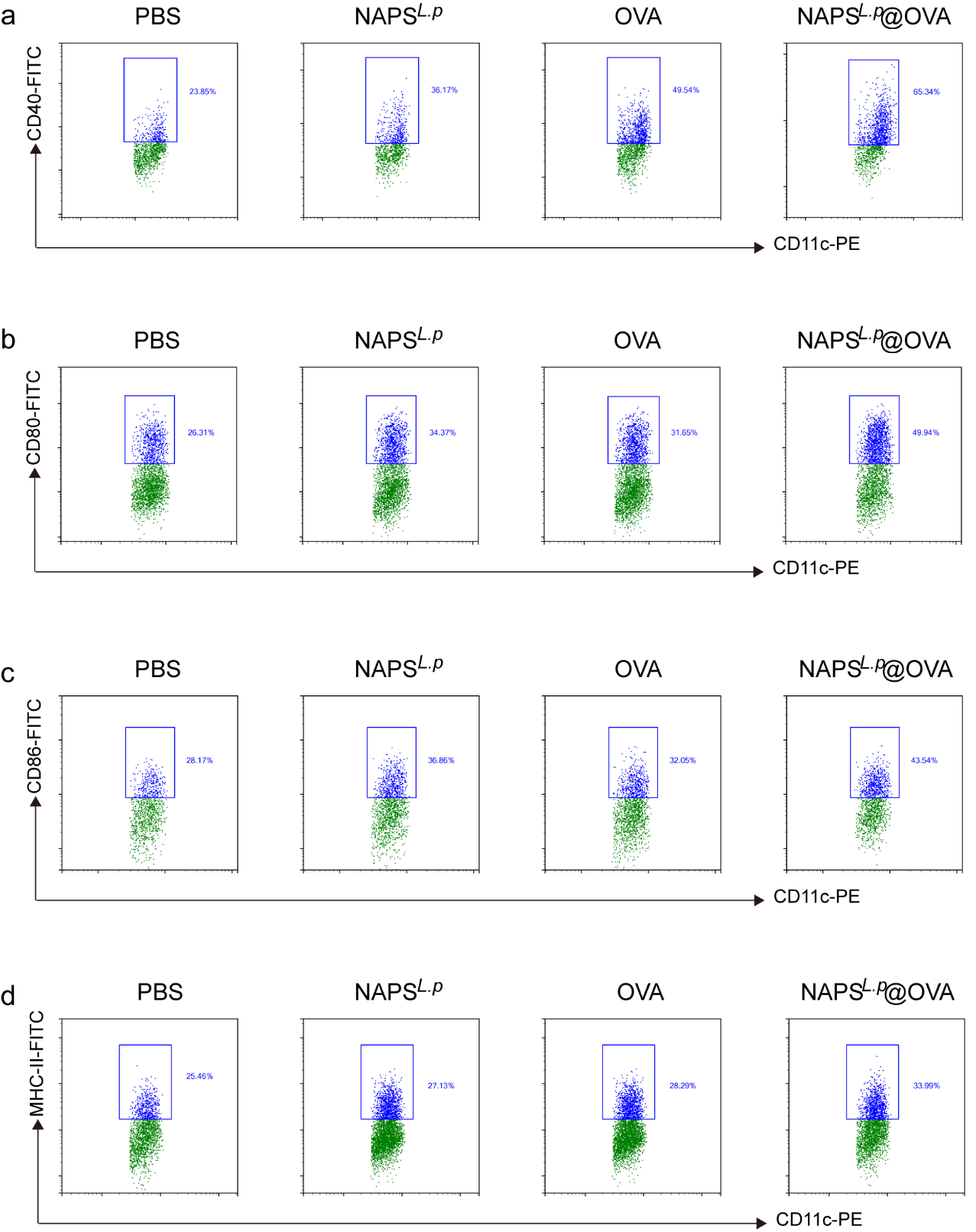
**

**Supplementary figure 18.** The expression levels of (a) CD40, (b) CD80, (c) CD86, and (d) MHC-II, were measured with flow cytometers. The data are presented as mean ± SD (n = 3).

**
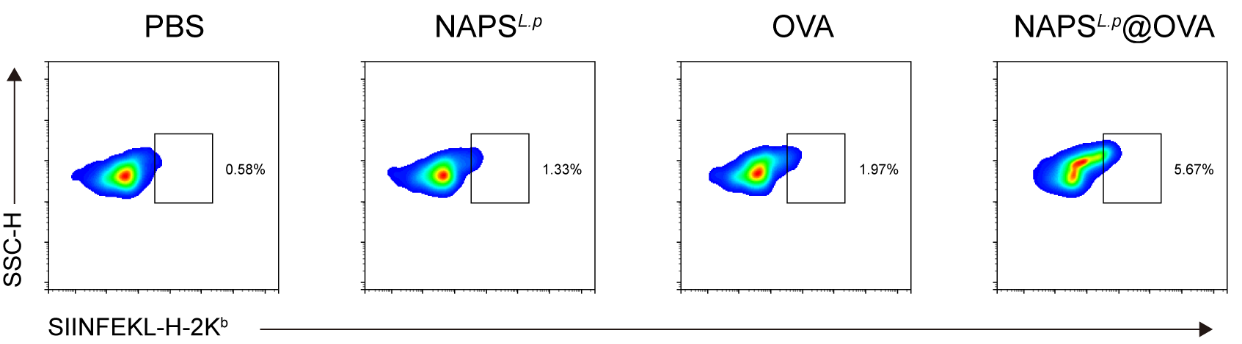
**

**Supplementary figure 19.** Representative flow cytometry plots display the proportion of SIINFEKL-H-2K^b+^ BMDCs in various groups. The data are presented as mean ± SD (n=3).

**
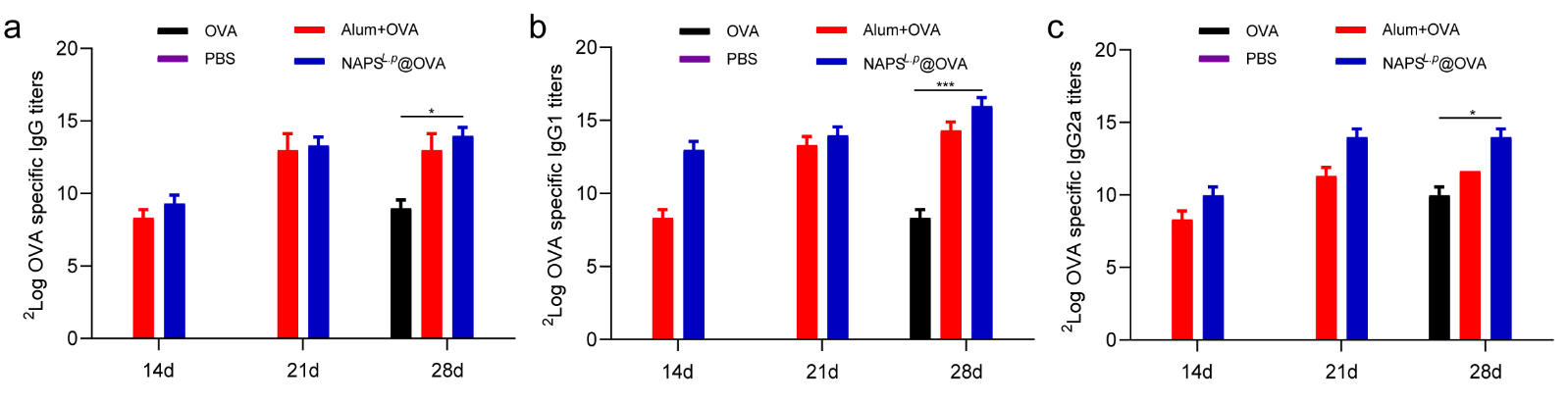
**

**Supplementary figure 20.** The titer of specific IgG and IgG subclass in mice serum (A) OVA-specific serum IgG antibodies of mice on days 14, 21, and 28 after the first immunization; (B) OVA-specific serum IgG1 antibodies of mice on days 14, 21, and 28 after the first immunization; (C) OVA-specific serum IgG2a antibodies of mice on days 14, 21, and 28 after the first immunization. The data are presented as mean ± SD (n = 3). *p< 0.05; **p< 0.01; ***p< 0.001.

**
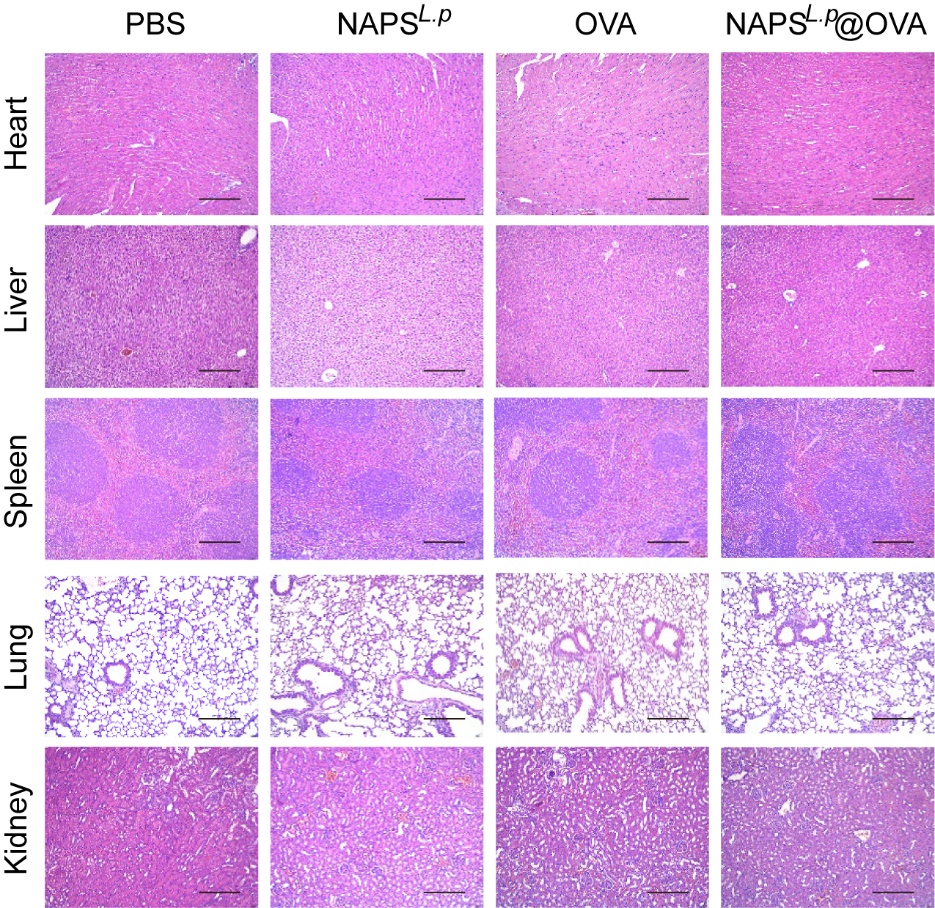
**

**Supplementary figure 21.** Pathological examination of organs from immunized mice by HE staining. Magnification 200×. Data are represented as the mean ± SD (n = 3).


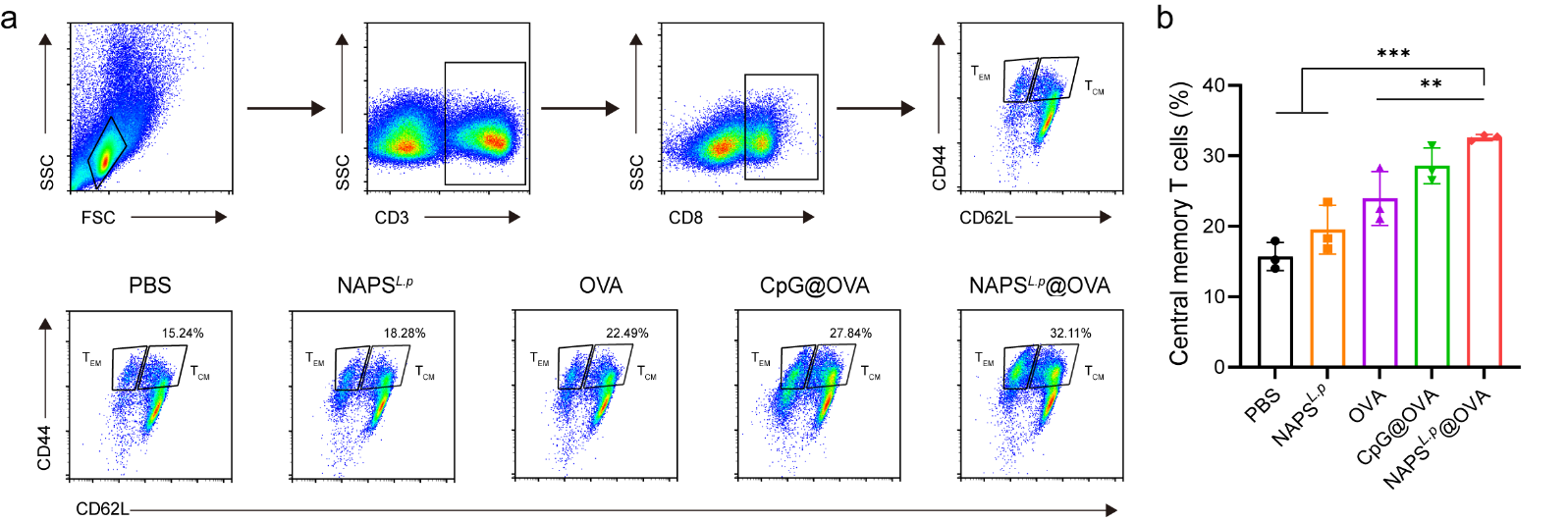


**Supplementary figure 22.** Analysis of immune memory in vivo. (a). Representative scatter plots and gating information derived from analysis of central memory T cells (T_CM_, CD3^+^ CD8^+^ CD44^+^ CD62L^+^) cells in Spleen. (b). Quantitative analysis of T_CM_ cells in Spleen (n=3). **, p < 0.01, ***, p < 0.001.


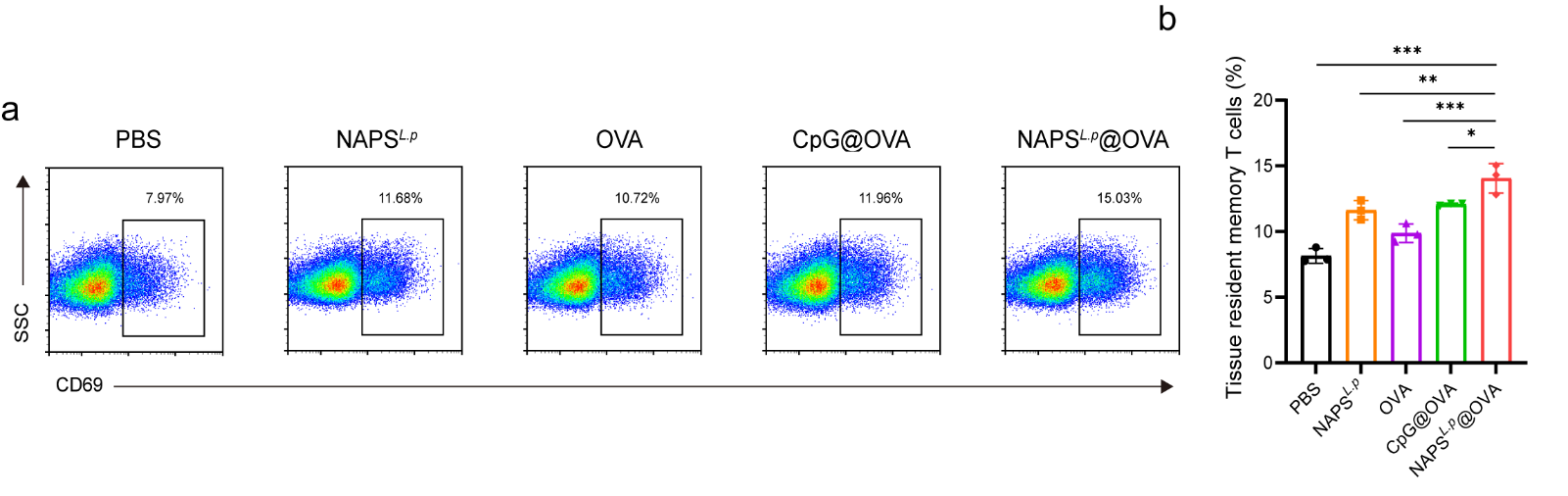


**Supplementary figure 23.** Analysis of immune memory in vivo. (a). Representative scatter plots and gating information derived from analysis of tissue resident memory T cells (T_RM_, CD3^+^ CD69^+^) cells in Spleen. (b). Quantitative analysis of T_RM_ cells in Spleen (n=3). *, p < 0.05, **, p < 0.01, ***, p < 0.001.


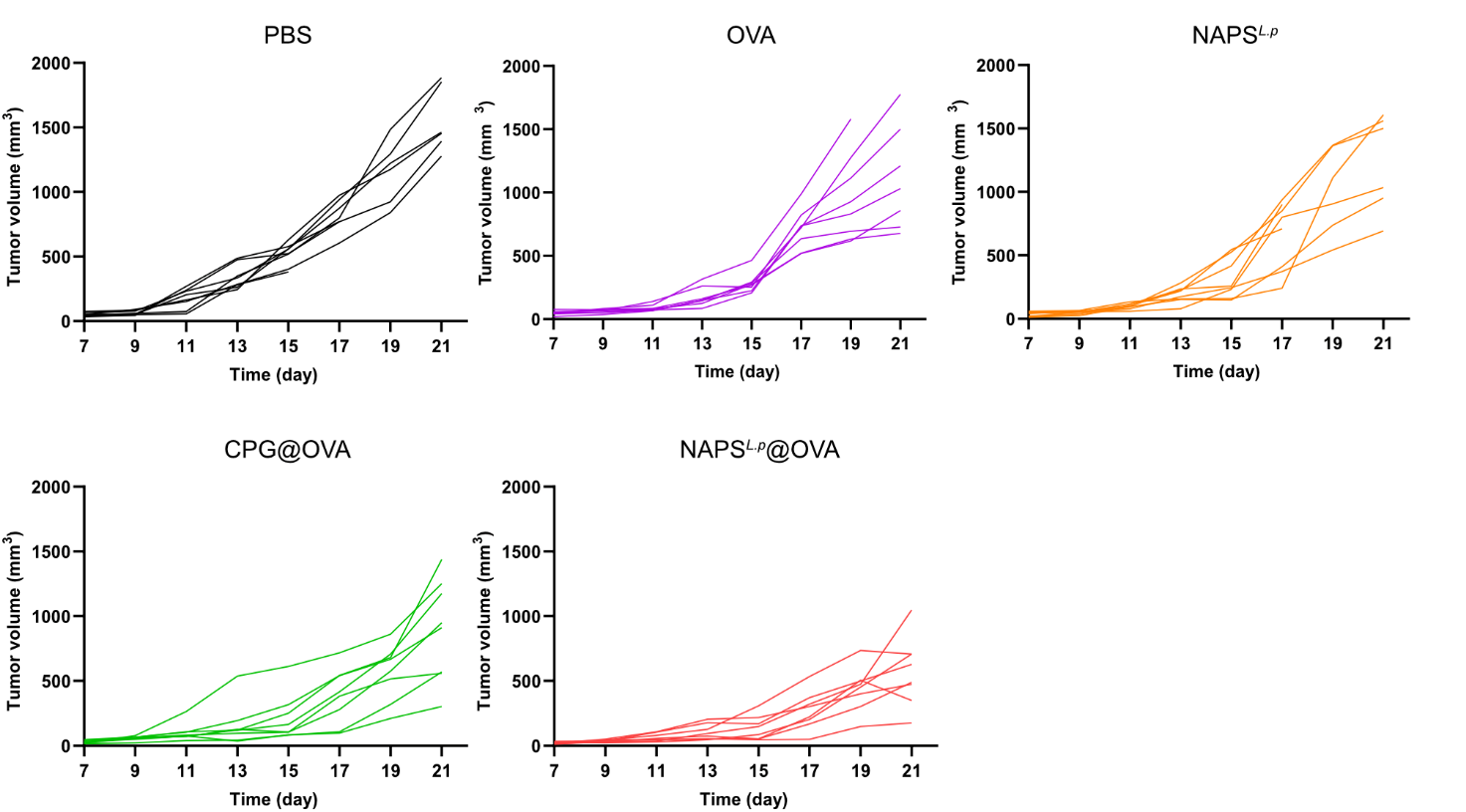


**Supplementary Figure 24.** Growth curves of subcutaneously implanted B16-OVA tumors in each mouse were recorded every other day from day 7 to day 21 (n = 8).

**Supplementary figure 25.** Body weight changes of mice in different groups (n = 8).


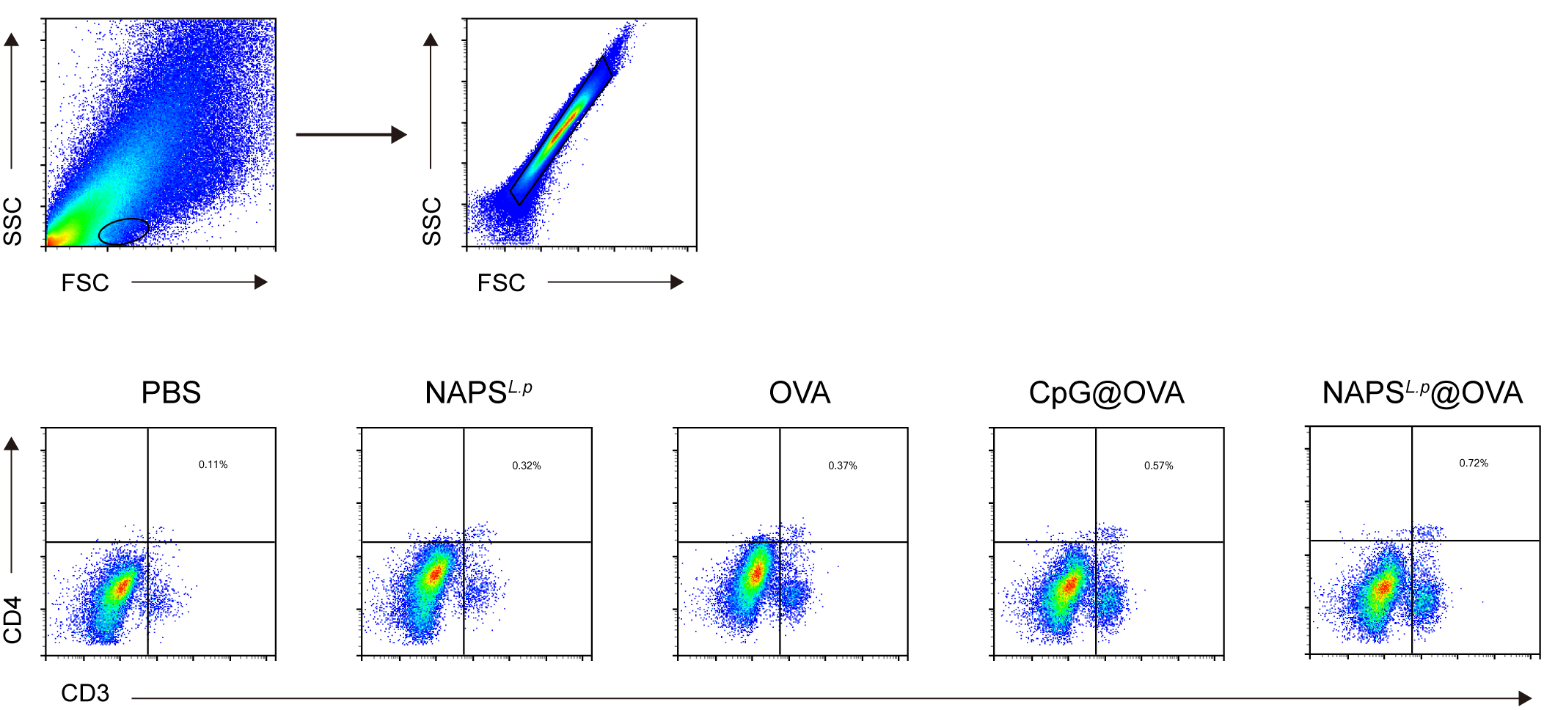


**Supplementary figure 26.** Representative scatter plots and gating information derived from analysis of CD4^+^ T cells in tumor tissue.


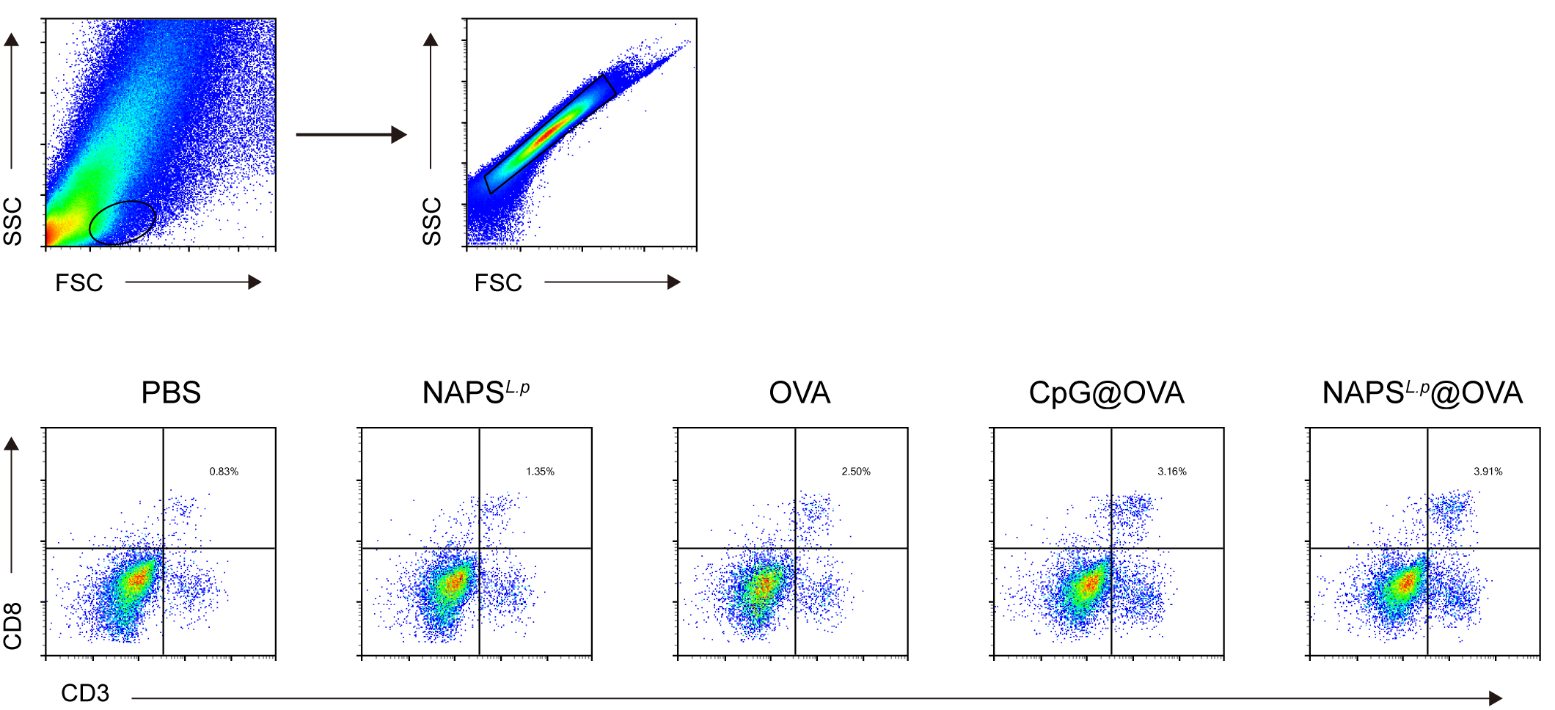


**Supplementary figure 27.** Representative scatter plots and gating information derived from analysis of CD8^+^ T cells in tumor tissue.


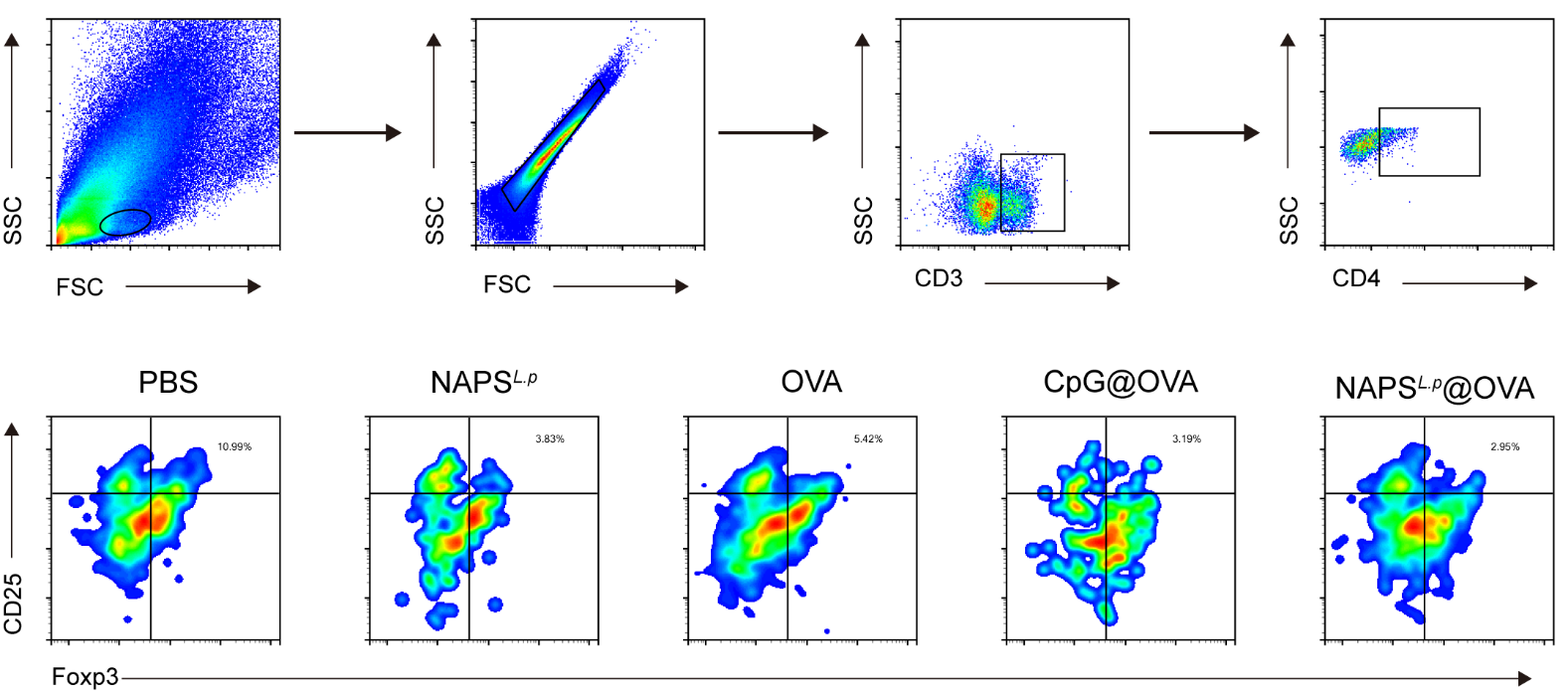


**Supplementary figure 28.** Representative scatter plots and gating information derived from analysis of Tregs T cells in tumor tissue.


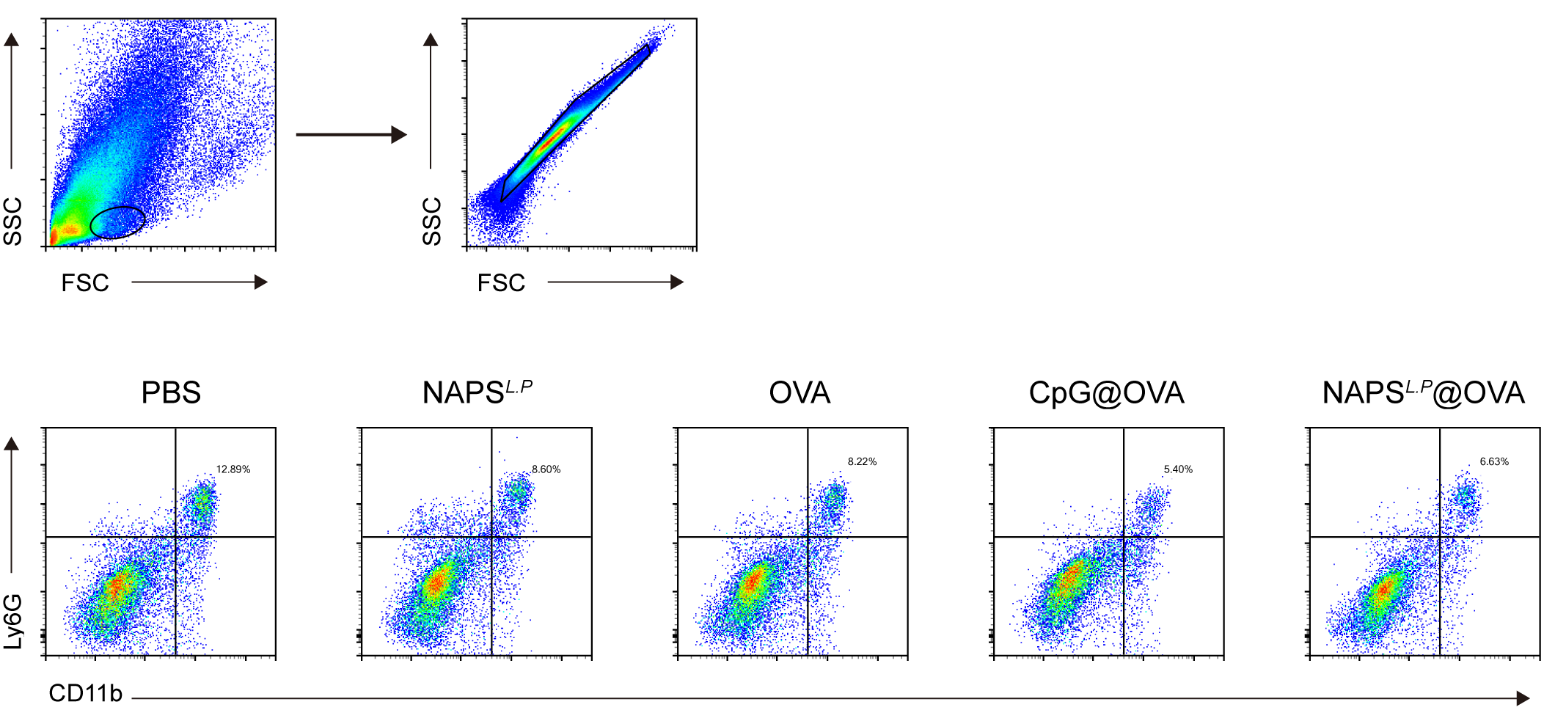


**Supplementary figure 29.** Representative scatter plots and gating information derived from analysis of MDSCs in tumor tissues.


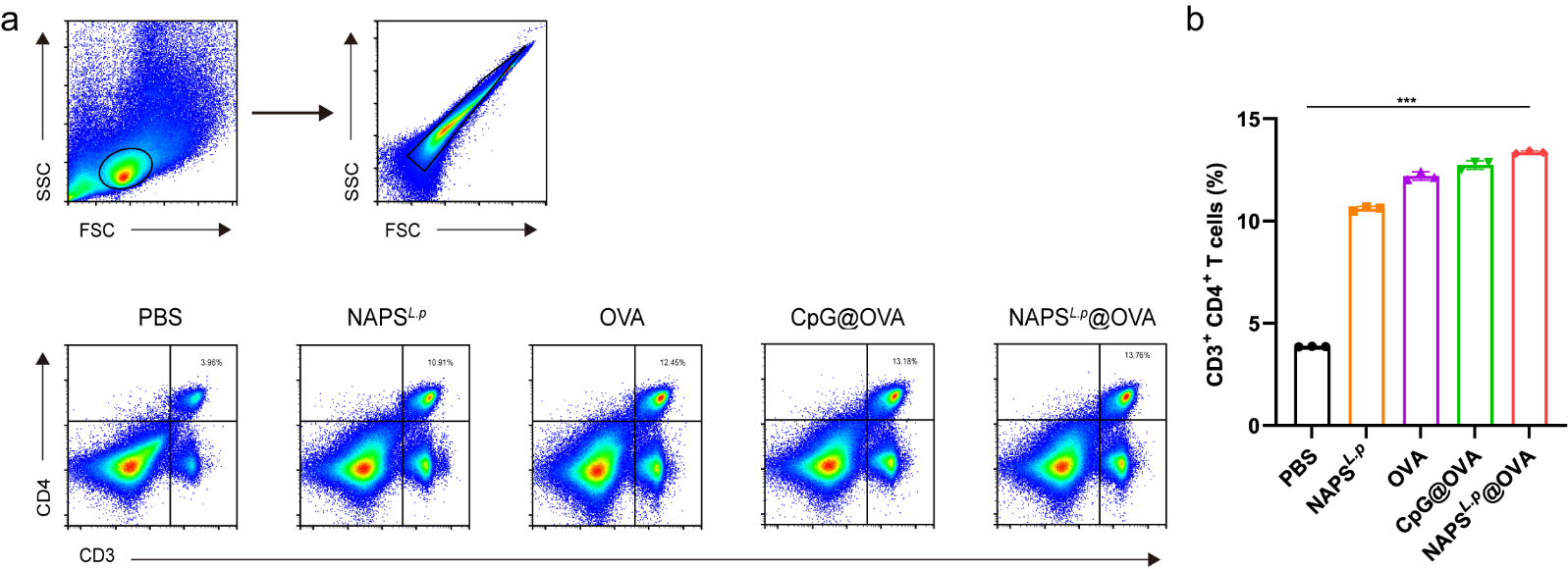


**Supplementary figure 30.** (a). Representative scatter plots and gating information derived from analysis of CD4^+^ T cells in Spleen. (b). Quantitative analysis of CD3^+^ CD4^+^ T cells in Spleen (n=3). ***, p < 0.001.


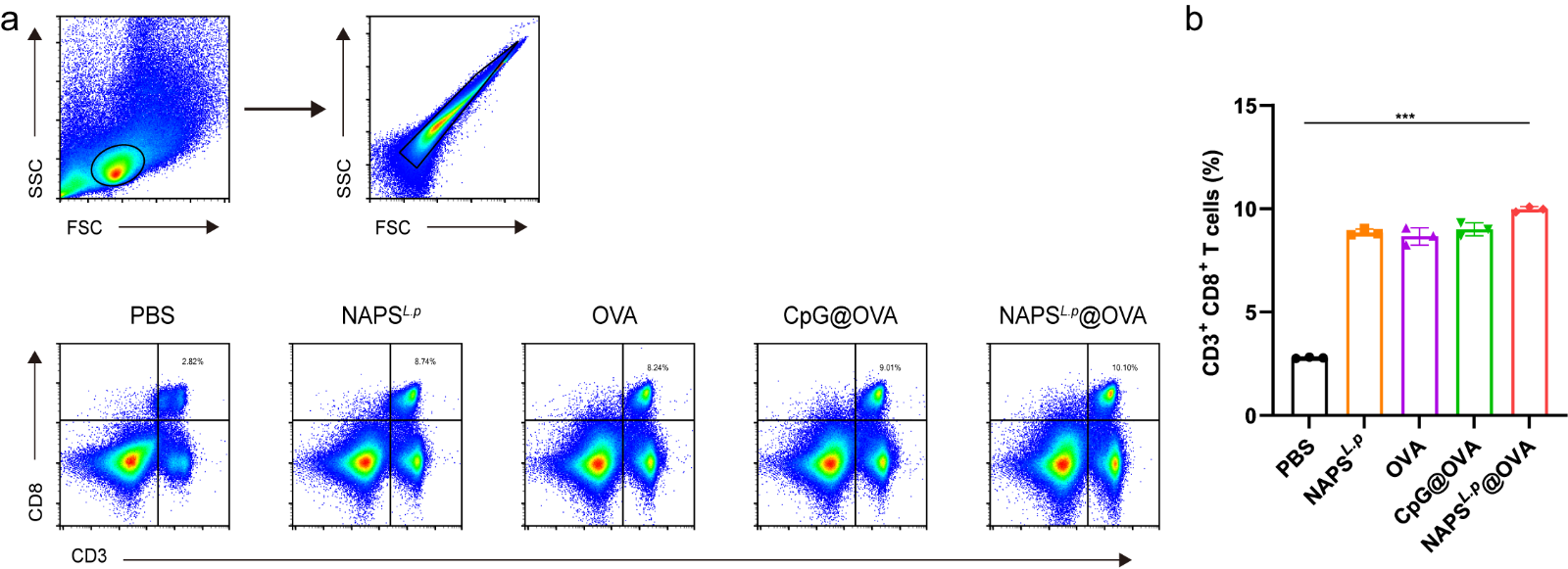


**Supplementary figure 31.** (a). Representative scatter plots and gating information derived from analysis of CD8^+^ T cells in Spleen. (b). Quantitative analysis of CD3^+^ CD8^+^ T cells in Spleen (n=3). ***, p < 0.001.


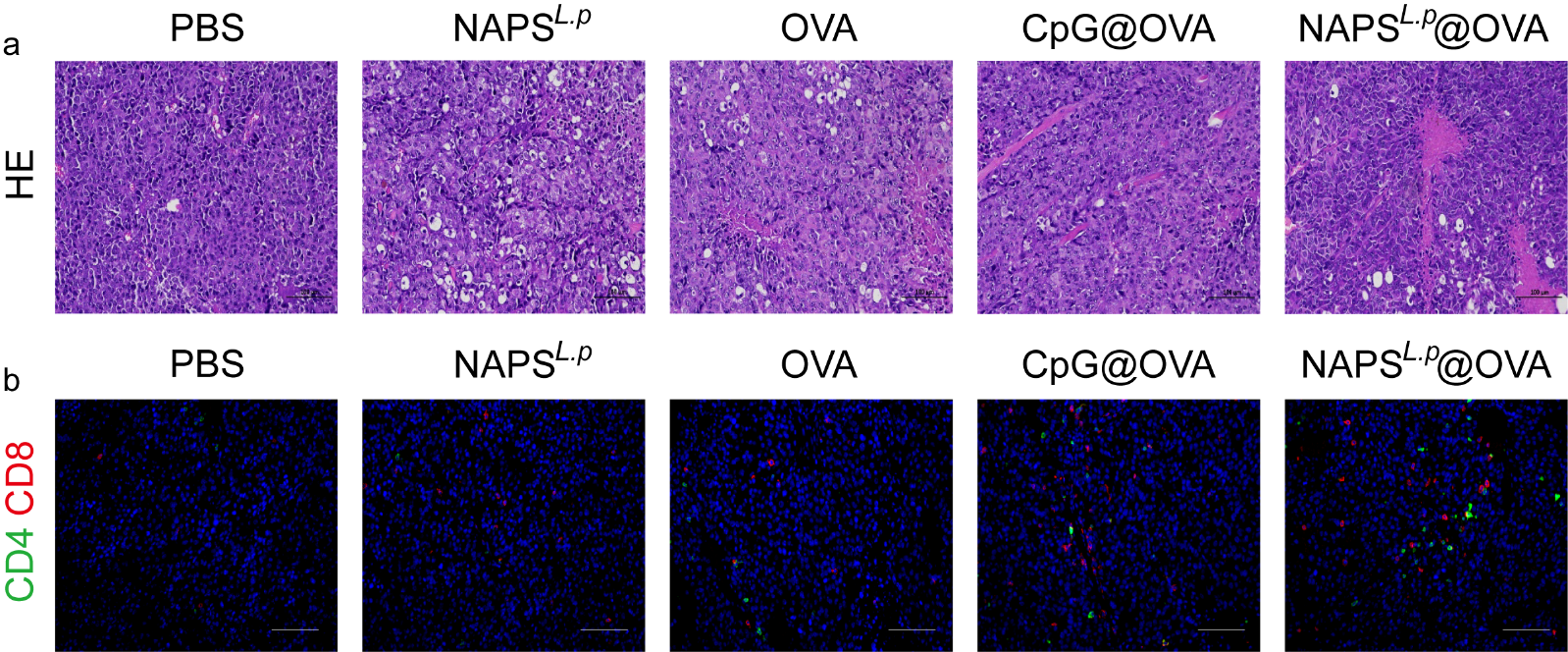


**Supplementary figure 32.** (a). H&E staining for pathological analysis and (b) immunofluorometric assays for showing the levels of CD4 and CD8 in tumors after vaccinations. The data are presented as mean ± SD (n = 3).


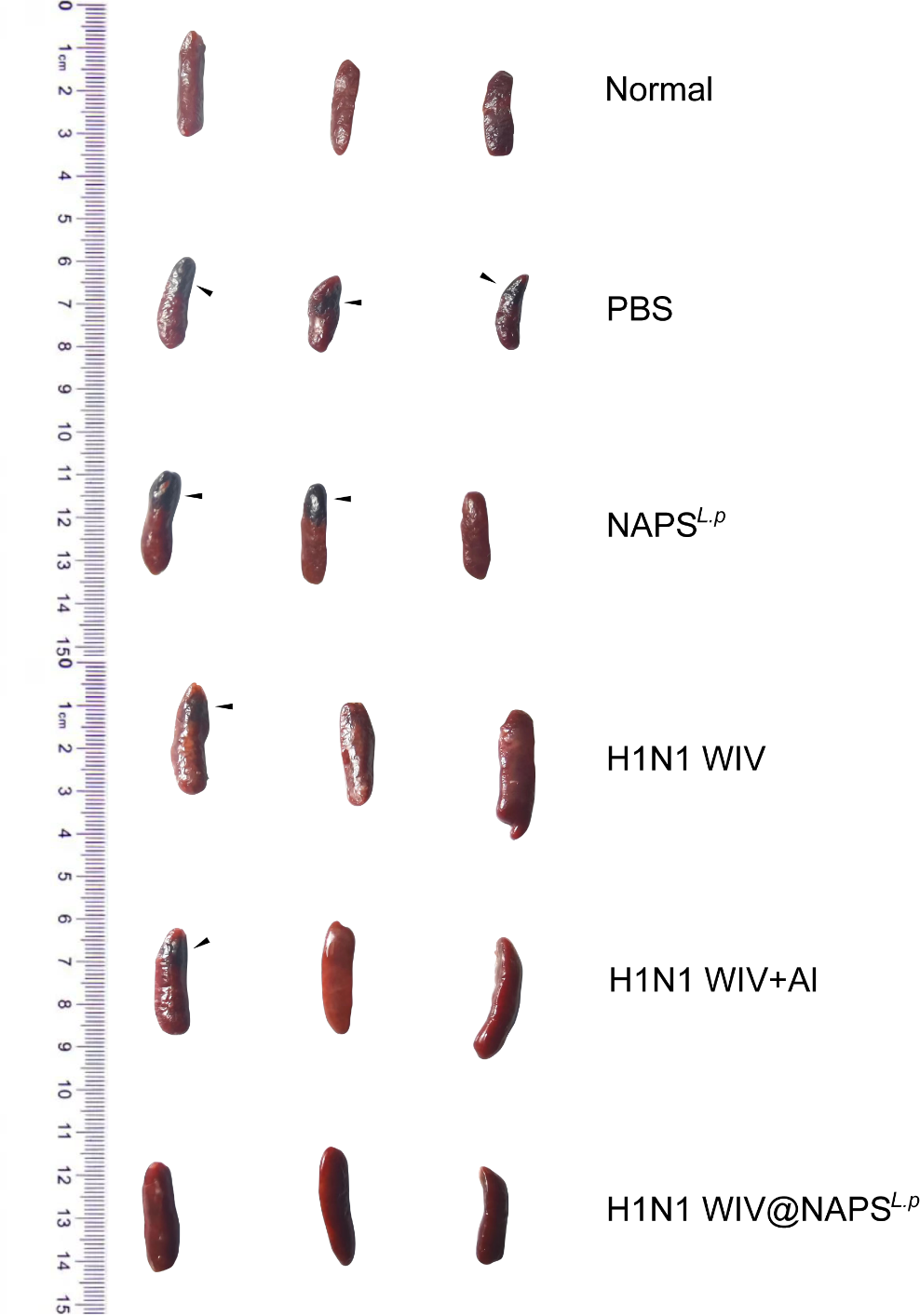


**Supplementary figure 33.** The pathological change of murine spleens post challenge. The vaccinated mice were challenged with 10^6^ PFU of the H1N1 virus 28 days post primary immunization. The pathological changes in spleen (n=3/group) on day 5 p. i.
